# Cell signaling heterogeneity is modulated by both cell-intrinsic and -extrinsic mechanisms: An integrated approach to understanding targeted therapy

**DOI:** 10.1371/journal.pbio.2002930

**Published:** 2018-03-09

**Authors:** Eunjung Kim, Jae-Young Kim, Matthew A. Smith, Eric B. Haura, Alexander R. A. Anderson

**Affiliations:** 1 Integrated Mathematical Oncology Department, Moffitt Cancer Center and Research Institute, Tampa, Florida, United States of America; 2 The Department of Thoracic Oncology, Moffitt Cancer Center and Research Institute, Tampa, Florida, United States of America; 3 Graduate School of Analytical Science and Technology (GRAST), Chungnam National University, Daejeon, Republic of Korea; Institute for Systems Biology, United States of America

## Abstract

During the last decade, our understanding of cancer cell signaling networks has significantly improved, leading to the development of various targeted therapies that have elicited profound but, unfortunately, short-lived responses. This is, in part, due to the fact that these targeted therapies ignore context and average out heterogeneity. Here, we present a mathematical framework that addresses the impact of signaling heterogeneity on targeted therapy outcomes. We employ a simplified oncogenic rat sarcoma (*RAS*)-driven mitogen-activated protein kinase (MAPK) and phosphoinositide 3-kinase-protein kinase B (PI3K-AKT) signaling pathway in lung cancer as an experimental model system and develop a network model of the pathway. We measure how inhibition of the pathway modulates protein phosphorylation as well as cell viability under different microenvironmental conditions. Training the model on this data using Monte Carlo simulation results in a suite of in silico cells whose relative protein activities and cell viability match experimental observation. The calibrated model predicts distributional responses to kinase inhibitors and suggests drug resistance mechanisms that can be exploited in drug combination strategies. The suggested combination strategies are validated using in vitro experimental data. The validated in silico cells are further interrogated through an unsupervised clustering analysis and then integrated into a mathematical model of tumor growth in a homogeneous and resource-limited microenvironment. We assess posttreatment heterogeneity and predict vast differences across treatments with similar efficacy, further emphasizing that heterogeneity should modulate treatment strategies. The signaling model is also integrated into a hybrid cellular automata (HCA) model of tumor growth in a spatially heterogeneous microenvironment. As a proof of concept, we simulate tumor responses to targeted therapies in a spatially segregated tissue structure containing tumor and stroma (derived from patient tissue) and predict complex cell signaling responses that suggest a novel combination treatment strategy.

## Author summary

A signaling pathway is a network of molecules in a cell that is typically initiated by stimuli (e.g., microenvironmental cues) acting on receptors and internal signaling molecules to determine cell fate. Signaling pathways in cancer cells are different from those in normal cells, and this difference helps cancer cells to grow and thrive indefinitely. Drugs that target the aberrant signaling pathways in cancer cells (often referred to as targeted therapy) are promising for improving treatment outcomes of many different cancers in patients. However, most patients eventually develop resistance to these drugs. Resistance may already be present in the tumor or may emerge via mutation or via microenvironmental mediation. Tumor heterogeneity, which is characterized by subtle or dramatic differences among tumor cells, plays a key role in the development of drug resistance. Some tumor cells respond well to therapy, while others may adapt to the stress induced by the drug within the microenvironment. Moreover, removal of drug-sensitive cells may result in the competitive release of drug-resistant cells. Here, we present mathematical models to assess the impact of heterogeneity in signaling pathways within tumor cells on the outcomes of targeted therapy. We consider a simplified version of two well-known signaling pathways that modulate the growth of lung cancer cells. By using different targeted therapies, we quantify the effect of pathway inhibition on protein activity and cell viability and developed a mathematical model of the network, which is trained to reproduce these data and to develop a panel of heterogeneous in silico cells. The model predicts potential mechanisms of drug resistance and proposes combination therapies that are effective across the panel. We validate these combination therapies experimentally using the lung cancer cells and integrated the in silico cells into a computational lung tissue model that explicitly captures the microenvironment of lung cancer. Our results suggest that heterogeneity in both the tumor and microenvironment impacts treatment response in different ways and suggest a novel combination therapy for a better response.

## Introduction

Normal cell signaling is significantly altered in cancer as a result of genetic and epigenetic changes, facilitating uncontrolled proliferation and cell survival [1, 2]. Targeted therapies directly exploit these alterations by blocking the activity of specific proteins typically mutated or abnormally up-regulated [3]. These therapies have elicited dramatic success in controlling the growth of multiple cancers [4–10] but showed little to moderate impact on others [11, 12]. Drug resistance, however, remains a major problem due to both cancer cell–intrinsic (innate and acquired) resistance mechanisms [13] and microenvironment-mediated resistance [14–16].

Tumor heterogeneity is known to contribute to drug resistance [17, 18]. Cancer cells within a tumor exhibit differential genetic and phenotypic characteristics [19]. Genomic heterogeneity leads to cell-to-cell variability in protein expression and activity as genes drive the production of proteins. Protein activity is variable even in genetically identical cancer cell populations in the same microenvironment [20–22]. This cell-to-cell variability arises from intrinsic stochastic fluctuations [23–30] and variation in microenvironmental conditions that affect the protein-signaling network. This variation can affect sensitivity to stimuli, contribute to cell phenotype decisions, and cause clonal cells to differently respond to stimulus and targeted therapies (e.g., erlotinib) [31, 32].

Understanding how cancer cell signaling variation affects targeted therapy outcomes is challenging. Cancer-driven signaling proteins do not function in isolation but rather function in protein complexes that belong to large and complex signaling networks that govern key phenotypic processes such as proliferation, apoptosis, and response to targeted therapy [33, 34]. Furthermore, the cancer signaling network and drug response are modulated by microenvironmental factors [35, 36]. Therefore, experimental data obtained from simplified cell-based experiments in single uniform environments will have limited ability to tease apart the impact of signaling network and microenvironmental variation on targeted therapy outcomes. A number of previous studies have used mathematical models to understand complex signaling networks and improve treatment strategies. Various modeling approaches were developed (reviewed in [37]). Boolean or probabilistic Boolean models were developed to analyze cancer signal pathways and predict treatment outcomes [38–41]. A logical modeling approach was employed to understand various cell signaling pathways [42–47]. An artificial neural network approach was used to map between microenvironments, pathways, and phenotypes [48]. Detailed kinetic models of cell signaling pathways have been studied using systems of ordinary differential equations (ODEs) [49–57]. Most studies, however, ignore signaling heterogeneity or extrinsic variation in microenvironmental cues that will differentially stimulate the signaling network.

**Fig 1:**
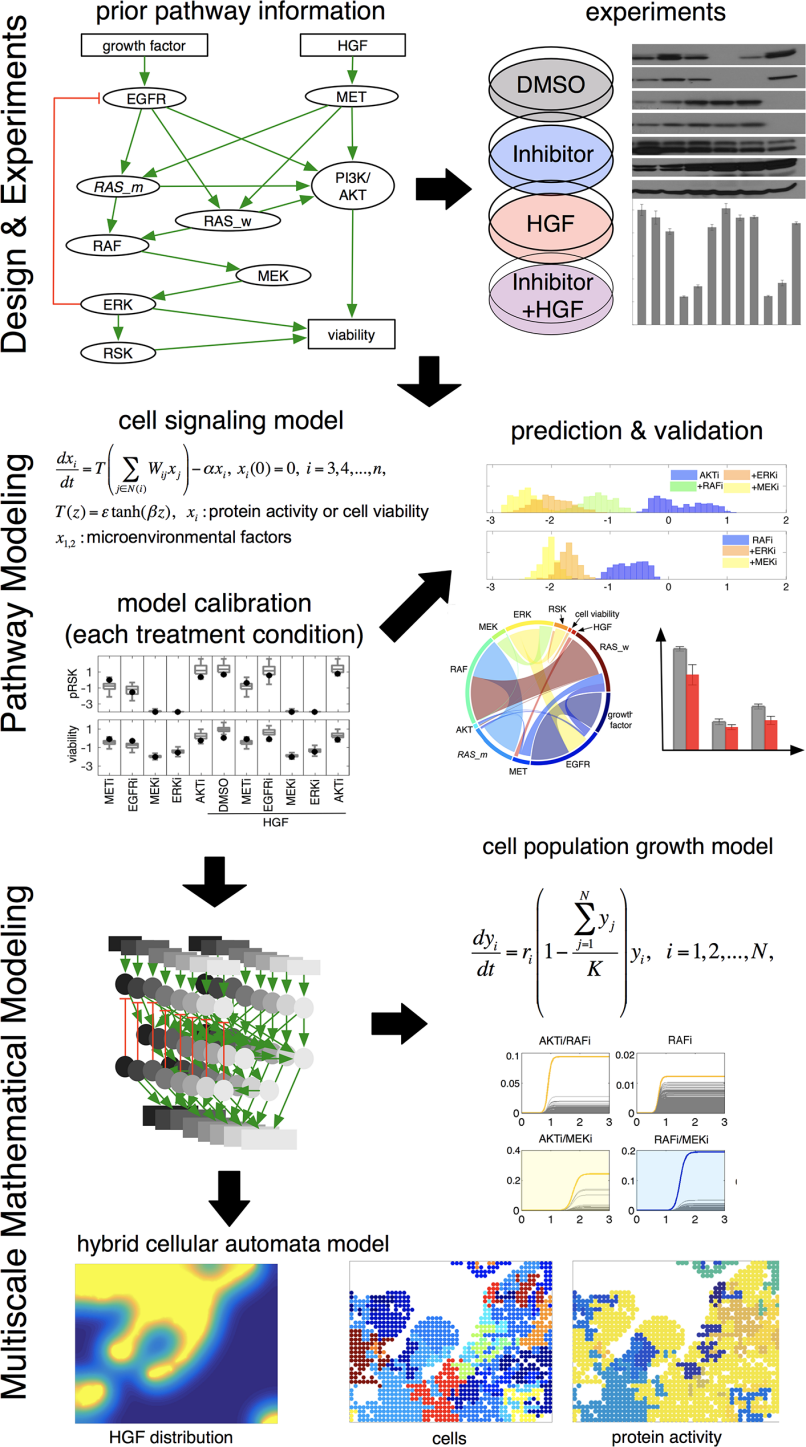
Overview. A simplified signaling pathway was derived based on literature. Various kinase inhibitors were applied to an experimental model system to perturb the simplified pathway in different microenvironmental conditions (e.g., HGF and no-HGF). A mathematical model of the pathway was developed and calibrated by comparing model predictions with experimental data. The model predictions were validated with experimental data. A validated pathway model was integrated into a logistic growth model to simulate the impact of competition between cells in a homogeneous and resource-limited microenvironment. Another integration of the pathway model into a hybrid model highlighted the impact of both microenvironmental heterogeneity and direct spatial competition of one cell with its direct neighbors on targeted treatment outcomes. AKT (PKB), protein kinase B; DMSO, Dimethyl sulfoxide (control); EGFR, epidermal growth factor receptor; ERK, extracellular receptor kinase; HGF, hepatocyte growth factor; MEK, Mitogen-activated protein kinase kinase; MET (c-MET), tyrosine-protein kinase Met or hepatocyte growth factor receptor (HGFR); PI3K, phosphoinositide 3-kinase; RAF, rapidly accelerated fibrosarcoma; RAS, rat sarcoma; RAS_m, mutated RAS; RAS_w, wild-type RAS; RSK, ribosomal S6 kinase.

To investigate the effects of signaling heterogeneity on targeted therapy outcomes, we develop an integrated approach combining in vitro experiments with three different mathematical models, an intracellular signaling model, a cancer cell population growth model, and a hybrid cellular automata (HCA) model of tumor and stroma (see Fig 1 for an overview).

## Materials and methods

### Mathematical modeling of MAPK and PI3K-AKT pathway

**Fig 2:**
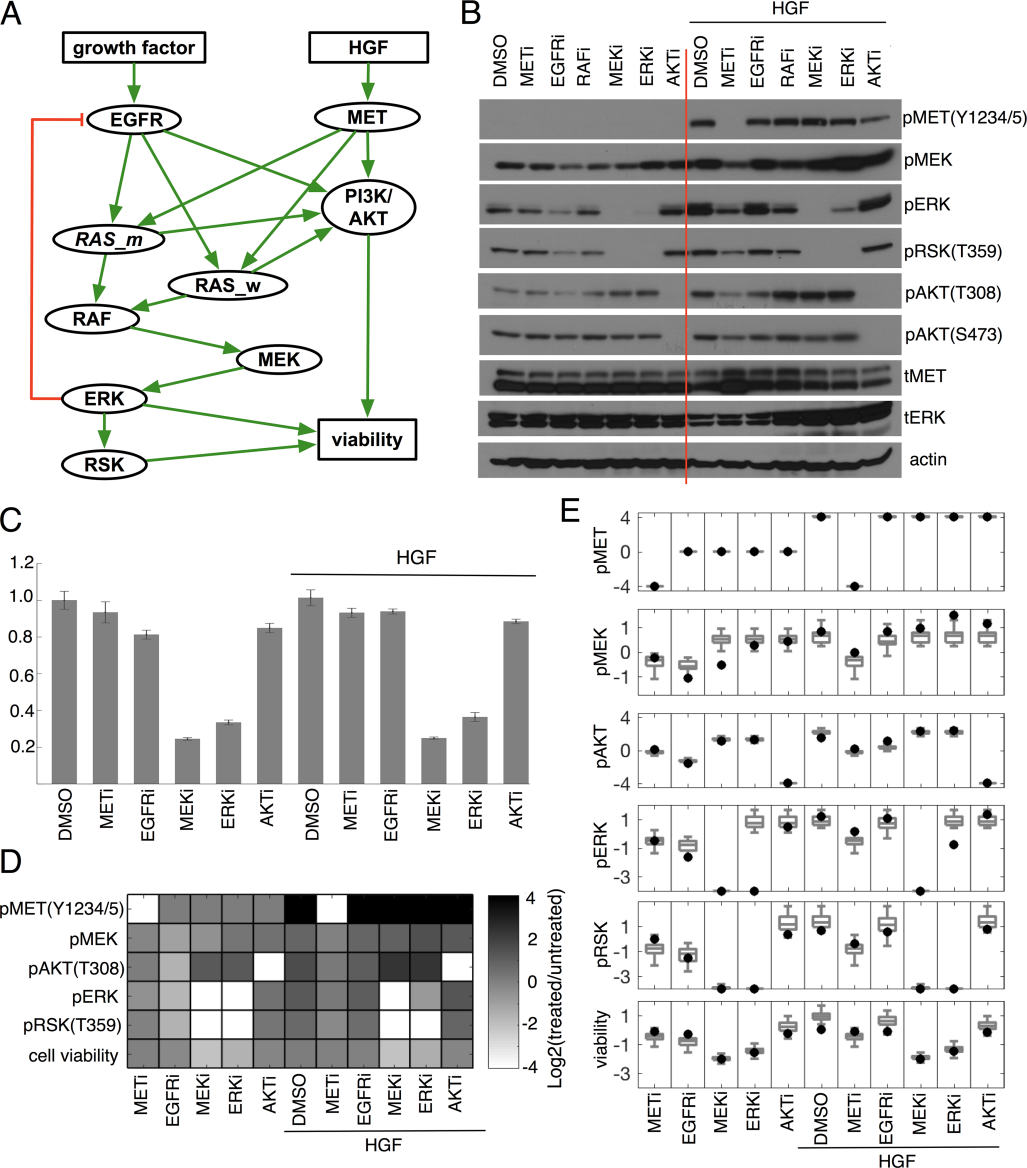
Signaling pathway model development and model calibration. (A) Simplified Signaling Network Model. Two inputs (growth factor and HGF), signaling protein nodes (EGFR, MET, *RAS_m*, RAS_w, PI3K/AKT, RAF, MEK, ERK, RSK), and one output (cell viability). Of note, *RAS_m* indicates a mutant *RAS*, while RAS_w indicates a wild-type RAS. A green line represents a positive relation (stimulation) and red line represents a negative relation (inhibition). (B) pMET (Y1234/5), pMEK, pERK, pAKT (both T308 and S473), and pRSK (T359) expression after different inhibitors (1 μM), MET inhibitor (METi, PHA665752), EGFR inhibitor (EGFRi, Erlotinib), RAF inhibitor (RAFi, LY3009120), MEK inhibitor (MEKi, GDC0623), ERK inhibitor (ERKi, SCH772984), and AKT inhibitor (AKTi, MK2208) in both control medium (DMSO) and after 2-hour stimulation-by-HGF (50 ng/mL) condition (DMSO plus HGF). (C) Relative cell viabilities after treatments. Cells were treated inhibitors (1 μM) for 72 hours. Cell viabilities were assessed by CellTiter-Glo assay (Promega). Representative triplicates (± SD) are presented, which showed similar results at least three times. (D) The western blots were quantified using ImageJ and relative changes (log2 scale) are reported. Average values of relative cell viabilities are also reported in log2 scale. All the data are normalized to the treatment-naïve control condition. pAKT (T308) readouts are quantified and used in the model. Of note, we didn’t quantify total protein levels because our primary interest was protein activity (protein phosphorylation). The effects of all inhibitors are modeled by assuming very small activity of a target protein (i.e., 1/16 of control, Methods section), and therefore pMET under METi is set be a very small number (i.e., 1/16 of control) for consistency. (E) Comparison between model predictions (gray box plots) and experimental data (black dots). A log2 fold change of pMET, pMEK, pAKT, pERK, pRSK, and cell viability after treatments of different inhibitors (METi, EGFRi, MEKi, ERKi, AKTi) in both a control medium and HGF-stimulated conditions. RMSE of each protein is following. pMET: 0.03; pMEK: 0.49; pAKT:0.33; pERK: 0.96; pRSK: 0.57; and cell viability: 0.47. The numerical data used in Fig 2 are included in the first sheet S1 Data. AKT (PKB), protein kinase B; DMSO, Dimethyl sulfoxide (control); EGFR, epidermal growth factor receptor; ERK, extracellular receptor kinase; HGF, hepatocyte growth factor; MEK, mitogen-activated protein kinase kinase; MET (c-MET), tyrosine-protein kinase Met or hepatocyte growth factor receptor (HGFR); PI3K, phosphoinositide 3-kinase; RAF, rapidly accelerated fibrosarcoma; RAS, rat sarcoma; RAS_m, mutated RAS; RAS_w, wild-type RAS; RMSE, root-mean-squared-error; RSK, ribosomal S6 kinase.

We consider a simplified signaling network composed of interactions between key proteins in an oncogenic rat sarcoma (*RAS*)-driven mitogen-activated protein kinase (MAPK) and phosphoinositide 3-kinase (PI3K)–protein kinase B (AKT, PKB) pathway (Fig 2A). This pathway has been studied extensively, and the positive or negative feedback regulations between proteins in the pathway are known [58–62]. Of note, mammalian cells express three RAS gene family members (HRAS, NRAS, and KRAS), and our model is based on empirical data obtained from a lung cancer cell line (A549); KRAS is always activated by a point mutation (Gly12Ser), whereas the other two RAS proteins (HRAS and NRAS) are wild type. Recent studies reported the importance of crosstalk between wild-type and mutant RAS proteins in cancers driven by oncogenic mutant RAS [59, 63, 64]. Therefore, we consider two different types of RAS, wild type (RAS_w) and mutant type (*RAS_m*). The network node connectivity is based on prior pathway information between signaling proteins [58–62]. Most interactions are feed-forward and positive (Fig 2A, green lines) except the one negative feedback regulation of epidermal growth factor receptor (EGFR) by extracellular receptor kinase (ERK) (Fig 2A, red line). While network connectivity is assumed fixed in the model, the strength of interactions is variable and is modeled using a weight matrix (**W**). Each element in the network (node *x*_*i*_) is updated by solving the following equation,

$$\begin{array}{l} \frac{dx_{i}}{dt}=T\left(\sum_{j\in N(i)}W_{ij}x_{j}\right)-\alpha x_{i},\;x_{i}(0)=0,\;i=3,4,\ldots ,n,\\ T(z)=\varepsilon \tanh(\beta z), \end{array}$$ (1)

where *x*_1,2_ correspond to two inputs (growth factor and hepatocyte growth factor [HGF]), and *χ*_3,4…,*n*_ correspond to the relative change of protein activities or cell viability due to an inhibitor with respect to untreated conditions (log_2_ (treated/untreated)) to be consistent with experimental data. Of note, all of the experimental measures in our study are relative values, normalized to the unperturbed condition. The absolute concentration or activity of signaling proteins as well as cell viability are difficult to acquire from experiments performed in our study, namely western blot experiments and cell viability measurement assays. Therefore, the weights in the model represent relative abundance or protein activities in treated conditions compared to treatment-naïve conditions. The model assumes that the rate of change of a variable is determined by the linear combination of neighboring nodes with corresponding weights. This additive linear function has successfully described protein reaction networks [54, 55, 65] although other functions such as Michaelis-Menten kinetics are viable options [51]. In the experiments we carried out, the microenvironmental conditions are growth factor and HGF. The growth factor (model variable *x*_1_) is always present, while HGF (model variable *x*_2_) is present in only some of the experimental conditions. In particular, the HGF is not present in the control condition. All experimental results are normalized to this control (no-HGF) condition. To represent these experimental conditions in the model, the input value *x*_1_ is set to a nonzero constant value (e.g., *x*_1_ ≡ *C*), while the variable *x*_2_ is set to be 0 or nonzero (e.g., *x*_2_ ≡ *C*) if HGF is present. We chose to set the parameter constant *C* to be 10. *N*(*i*) represents the neighborhood of a protein node *i* (a set of nodes connected to the node *i*), and *α* indicates a tendency to return to the untreated state. The transfer function *T* accounts for saturation effects, and the constants *ε* and *β* modulate amplitude and slope. In the model, we set *ε* to be 4.5 and *β* to be 0.5 to model a smooth sigmoidal behavior.

Drug inhibition is modeled by knocking out a corresponding protein activity. For example, if a drug inhibits a protein *x*_*i*_, then the variable *x*_*i*_ is set to be a very small number ($x_{i}=\log_{2}\;\left(\frac{x_{treated}}{x_{untreated}}\right)=-\mu ,\mu \gg 1$). It is worth noting that we modeled the effect of drugs tyrosine-protein kinase Met inhibitor (METi), EGFRi, and AKTi by inhibiting pMET, pEGFR, and pAKT activities, respectively. Two other drugs, mitogen-activated protein kinase kinase (MEKi), and ERKi, are modeled to inhibit pERK and pRSK, respectively (Fig 2B).

### Logistic tumor growth

We combine the pathway model to a logistic growth ODE model to simulate the growth of in silico cells as a well-mixed population in a resource-limited environment. The model is defined as follows,

$$\frac{dy_{i}}{dt}=r_{i}\left(1-\frac{\sum_{j=1}^{N}y_{j}}{K}\right)y_{i},\;i=1,2,\ldots N,$$ (2)

where *y*_*i*_ represents the number of in silico cell *i*, *r*_*i*_ is the cell intrinsic growth rate of the cell *i*, *N* is the total number of cell types, and *K* is the carrying capacity (set to be 1 billion). To model influence of the signaling pathway on cell population growth, we formulate a cell population growth rate, *r*_*i*_, as a function of cell viability solutions and the treatment-naïve growth rate (*r*_*i*_ ≡ *f* (*θ*_*i*_, *r*_0_); *θ*_*i*_: cell viability solution in linear scale not in log2 scale; *r*_0_: growth rate in an untreated control condition). Specifically, the cell viability for each cell type is obtained by fitting pathway model to our experimental data, as described in the Results section. The cell viability of cell type *i* (*θ*_*i*_) represents the number of cell type *i* that survived after being given therapy relative to an untreated control condition (i.e., *θ*_*i*_ = *P*_*i*_(*t*)/*P*_0_(*t*), *θ*_*i*_: cell viability, *P*_*i*,0_(*t*): number of cell type *i* at time *t* in a treated and untreated condition, respectively). To obtain a functional form for the growth rate, we make the following assumptions. We assume that a cell population initially grows exponentially $(P_{i}(t)=P_{i}(0)e^{r_{i}t},\;P_{0}(t)={P_{0}(0)e}^{r_{0}t},\;t<T$ for some time *T*). We also assume that the number of initial cell population is the same in a treated condition and in an untreated control condition (*P*_*i*_(0) = *P*_0_(0)). Then, cell viability is presented as a function of growth rates and time ($\theta_{i}=P_{i}(t)/P_{0}(t)=e^{r_{i}t}/e^{r_{0}t}$). Solving the function for growth rate *r*_*i*_, we obtain a functional form for the growth rate. We use the doubling time of A549 cells (22 hours from [66]) to obtain the treatment-naïve growth rate (*r*_0_ = 0.76 per day). We use our cell viability assay experimental time point (*t* = 3 days, described in Results section). Now, we have a constant growth rate of cell type *i* for each treatment condition (500 cells x 28 treatment conditions, total 14,000 growth rates, *r*_*i*_). Then, the ODE Eq (2) is solved to simulate a given treatment response of cell *i* over time. All of the in silico cells are solved simultaneously competing for limited resource (carrying capacity *K*).

### HCA tumor model

The pathway model is integrated into an HCA model [67, 68] to simulate treatment responses in a spatially heterogeneous microenvironment. The model has the following assumptions. In the HCA, the cells are defined as points on a two-dimensional lattice that also contains continuous concentration fields of microenvironmental factors, together representing a cross-section of tumor composed of cancer cells and stroma (50 cells x 38 cells). Here, we define the tumor and stroma region explicitly based on an image segmentation of lung adenocarcinoma tissue from a patient. The tumor region contains cancer cells. Each cancer cell contains the pathway model, as developed above (Fig 2A), that links the microenvironment to cell phenotypes. The model grid can contain any number of possible microenvironmental variables. For simplicity, however, we consider only growth factors and HGF. The growth factors are assumed to be constant in the domain. We explicitly model HGF dynamics in space and time using the following partial differential equation,

$$\frac{\partial H(x,t)}{\partial t}=D\nabla^{2}H(x,t)-\lambda H(x,t),$$ (3)

where *H*(**x**,*t*) represents the concentration of HGF at a lattice point **x** in tumor region and at time *t*. *D* represents the diffusion rate, and *λ* is a decay rate. The parameter values used in a simulation are given in the corresponding figure legend. The concentration of HGF is fixed to be a constant value (*H*(**x**,*t*) = *γ*) in the stromal region. A Neumann boundary condition ($\frac{\partial H}{\partial n}(x)=0$, normal derivative = 0) was imposed on the domain boundary. A Dirichlet condition (*H*(**x**,*t*) = *γ*) was imposed at the interface between tumor and stroma.

The steady state solution of Eq (3) is fed into one of the inputs (HGF) in the pathway model in each cell (Fig 2A). The pathway determines cell viability and controls three different phenotypes—proliferation, quiescence, and death—as defined by the rules summarized in the flowchart (S1 Fig). Each cell is allowed to execute only one phenotype per time step (day). Of note, the model considers only orthogonal neighbors (north, west, south, and east) for space to divide or move. Cells are not allowed to leave nor enter across the boundary and are thus confined within the domain. We assume that the distribution of HGF barely changes during HCA simulation. For example, if a cell divides into two daughter cells, this increased number of cells does not impact the HGF distribution because we assume the consumption of HGF by cells negligible and that cells do not produce HGF. Because HGF consumption is minimal and the HGF diffusion timescale (approximate seconds) is a lot shorter than cell division time scale (approximate day) and the model domain size is small (50 cells x 38 cells), any consumption would quickly be equilibrated to the steady state.

### Cell line

A549 lung adenocarcinoma cell line was maintained in RPMI 1640 medium supplemented with 10% FBS. Cells were confirmed to be free of mycoplasma using PlasmoTest (Invivogen, San Diego, CA).

### Western blots

Cells were washed with ice-cold PBS, and whole cell extracts were prepared using lysis buffer (0.5% NP-40, 50 mM Tris-Cl, pH 8.0, 150 mM NaCl, 1 mM EDTA) supplemented with protease inhibitor (Roche, Mannheim, Germany) and phosphatase inhibitor cocktail (Sigma-Aldrich, Carlsbad, CA). Whole-cell extracts were resolved on SDS-PAGE and transferred to nitrocellulose membrane. The membrane was blocked in 5% skim milk/PBST and then incubated in primary antibody at 4°C overnight. Bound antibodies were visualized by horseradish peroxidase-conjugated secondary antibodies and SuperSignal West Pico Chemiluminescent Substrate (Thermo Scientific, Waltham, MA). Primary antibodies used for our study were purchased from Cell Signaling Technology (Danvers, MA) (except for β-actin, which was from Sigma-Aldrich, St. Louis, MO).

### Cell viability measurement

Cells were plated on 96-well plate at 2,000 cells per well and then exposed to drugs for 72 hours. Cell viability was analyzed by CellTiter-Glo (Promega, Madison, WI) according to the manufacturer’s recommendations.

### Proximity ligation assays

Proximity ligation assays were performed as described using Duolink Far Red kit (Sigma-Aldrich, Carlsbad, CA) with antibodies to the following: EGFR (clone B38, Cell Signaling, Danvers, MA), GRB2 (clone 81, BD, San Jose, CA), and AF488-conjugated pan cytokeratin (clone Ae1/Ae3, eBioSciences, San Diego, CA). Tissue specimen was from a de-identified patient treated at Moffitt Cancer Center and was obtained via an institutionally approved protocol.

## Results

We followed the steps described in Fig 1 to investigate the effects of signaling heterogeneity on targeted therapy outcomes. First, we develop an intracellular signaling pathway model. We construct a simplified cancer signaling pathway based on prior information about the pathway, and we experimentally perturb the pathway using various kinase inhibitors in two different microenvironmental conditions (Fig 1 Design & Experiments). Among key microenvironmental factors, HGF has been shown to contribute to resistance in multiple Food and Drug Administration (FDA)-approved targeted therapy drugs [35, 36]. Therefore, we consider HGF as an additional microenvironmental stimulus. Then, we build a mathematical model of the cancer signaling pathway to predict both signaling and a phenotypic response (cell viability change due to a given therapy) to different inhibitors that target the pathway (Fig 1 prior pathway information). It is important to note that the model includes two microenvironmental factors (i.e., growth factor or HGF) and cell viability (Fig 1 prior pathway information) in addition to intracellular proteins (Fig 1 prior pathway information). Model parameters are calibrated using experimentally measured protein expression levels and cell viability after different inhibitors are applied under different microenvironmental conditions (Fig 1 Pathway Modeling, model calibration). Now using this panel of in silico cells with calibrated signaling networks, we predict distributional responses to different targeted therapies, reveal possible mechanisms of drug response and resistance, and propose combination therapy strategies that could deal with heterogeneity. The model predictions are then tested experimentally.

Next, we develop a logistic cancer cell population growth model to describe tumor growth in a homogeneous but resource-limited microenvironment (Fig 1 Multiscale Mathematical Modeling). The intrinsic growth rate of each cancer cell is estimated based on the treatment-naïve growth rate and cell viability obtained from the signaling pathway model calibration. We predict post-treatment cell population heterogeneity and average efficacy after continuous application of various inhibitors (both mono and combination therapies). Finally, we develop an HCA model to investigate the effects of spatially heterogeneous microenvironments on targeted therapy outcomes (Fig 1 Multiscale Mathematical Modeling). The model couples continuous microenvironmental factors with a discrete cell–based model. Each individual cell contains the trained network model that links microenvironment to its phenotype, which determines cell fate in a given condition. As a proof of concept, we simulate the response to an inhibitor in a section of tissue composed of both tumor cells and stroma. The model predicts complex cancer cell signaling responses and treatment outcomes, driven by both cell-intrinsic and -extrinsic mechanisms.

### Experimentally measured cancer cell response to various kinase inhibitors

Kirsten rat sarcoma (KRAS)-driven cancer treatment is an important clinical need that remains largely unmet due to limited targeted drug efficacy of key downstream effectors, including MAPK and PI3K-AKT pathways. We therefore choose the KRAS mutant non–small-cell lung cancer (NSCLC) cell line (A549 cell line) as our experimental model system. Using our simplified oncogenic KRAS signaling pathway (Fig 2A, Mathematical modeling section for an explanation of how we obtained this simplified pathway) as a guide, we pharmacologically inhibited individual proteins in the MAPK pathway (MET, EGFR, MEK, ERK inhibitors) and PI3K-AKT (AKT inhibitor) pathway in A549 cells in the both absence and presence of HGF. Drug-induced changes in the phosphorylation of pathway proteins, surrogates for protein activity, were measured by western blotting (Fig 2B). Cell viability was also assessed after 72 hours of drug treatment (Fig 2C). These experimental data were quantified using ImageJ (Fig 2D), and all data were normalized to the control experimental condition (treatment-naïve condition).

### Model calibration

The quantified changes (Fig 2D) and the Monte Carlo simulation were then employed as an optimization procedure to estimate model parameters (weights, *W*_*ij*_) that minimize our cost function. A network with lower cost represents the experimental data more accurately. The cost function is defined as follows,

$$C(W)={\sum_{i}^{n}\sum_{d}^{M}\left(\bar{x_{i,d}}-y_{d}\right)}^{2}+\sum_{j\in N(r)}\frac{\chi }{1+\exp(\eta w_{jr})}$$ (4)

where $\bar{x_{i,d}}$ is the steady state activity of protein or cell viability *x*_*i*_ in treatment condition *d*, *y*_*d*_ represents experimental data, and *M* is the total number of treatment conditions. The weight *w*_*jr*_ indicates the weight between *RAS_m* (*r*) and its neighbors (*N*(*r*)), and the constants (*χ*, *η*) modulate the magnitude of the penalty. The first term explains the difference between model prediction and experimental data for a network *W*. The second term is directed at the activating *RAS* mutation and incorporates a penalty for estimated weights from the *RAS_m* node (mutant RAS) that are too small. We included this penalty because our model is based on empirical data of a KRAS mutant cancer cell line (A549 cell), where the resulting KRAS protein is constitutively active. We aimed to capture this activating mutation by penalizing small weights from *RAS_m* to its neighbors.

We used the following method to implement Monte Carlo simulations:

1. Initialize a sparse weight matrix (*W*, *W*_*ij*_ = 0, for no connection in Fig 2A) with random numbers.
2. Enforce the weight elements to satisfy the prior pathway information (*W*_*ij*_ = |*W*_*ij*_| for green line; *W*_*ij*_ = −|*W*_*ij*_| for red line; Fig 2A).
3. Update protein node values using the Eq (1).
4. Evaluate cost *C*_*old*_ = *C* (*W*) using the Eq (4).
5. Randomly select an element of weight matrix and perturb it ($W_{ij}^{new}=W_{ij}+\eta ,\eta ∼N(0,\sigma ),$) for some small *σ*, *σ* > 0) and enforce weight constraints (step 2).
6. Update cost *C*_*new*_ = *C* (*W*^*new*^) (Eq [4]) with a perturbed weight matrix (*W*^*new*^).
7. If *C*_*new*_ < *C*_*old*_, accept the perturbation; otherwise, mostly reject the perturbation. Only accept the perturbation with a small probability of *p*, where *p* = exp(−*ν*(|*C*_*new*_ − *C*_*old*_|) for some large *ν*.
8. Go to the step 5 and repeat 5 through 7 until achieving error = |*C*_*new*_ − *C*_*old*_| < *δ*, where *δ* is a predefined tolerance (for small non-negative number, *δ* > 0).

The model calibration resulted in more than 5,000 weight matrices that fit to the experimental data. We selected the best 500 (top 10%) weight matrices and used these to define our 500 in silico cells. The distributions of in silico cells are presented as box plots in Fig 2E along with the experimental measures. Errors (root-mean-squared-error [RMSE] formula given in S1 Text) are in the range of (0.03–0.56, except ERK: 0.96). The fit of ERK was poor because of unexpected inhibition of pERK by the drug (ERK inhibitor, SCH772984) [69]. The trained networks (weights) are quite heterogeneous (S1 Table). The distribution for each weight is different (S1 Table, skewed, normal, bimodal distributions, with a range of heterogeneity [Shannon] index values).

The weights here may represent relative protein abundance or protein-binding activity. There is ample evidence for differential abundance of protein species across cellular populations. An excellent example was recently published showing that variations in adaptor protein abundance are a major source of regulation of the EGFR-MAPK pathway [70]. There are several examples of differential binding activity of proteins in cell signal transduction. It is well established that adaptors such as GRB2, SHC1, and GAB1 can be recruited to receptor tyrosine kinases (RTKs) either directly or indirectly. Therefore, stochastic variation in multiprotein complex composition at individual receptors exists, and this will vary both within and between cells. It is also accepted that activation-induced receptor degradation and phosphatase activity will affect not only RTK adaptor interactions but also downstream signaling molecules such as rapidly accelerated fibrosarcoma (RAF), MEK, and ERK. Additionally, we have previously shown that, in *EGFR* mutant cell lines, only a fraction of the receptor is phosphorylated, and cell lines harboring the same oncogenic mutation have different levels of phosphorylated Tyrosine (Tyr) and Serine/threonine (Ser/Thr) residues [71]. Collectively, these examples indicate that protein–protein interactions in response to growth factors are not simply on–off states and that multiple factors independent of protein abundance control final signaling output.

### Distributional responses to kinase inhibitors

**Fig 3:**
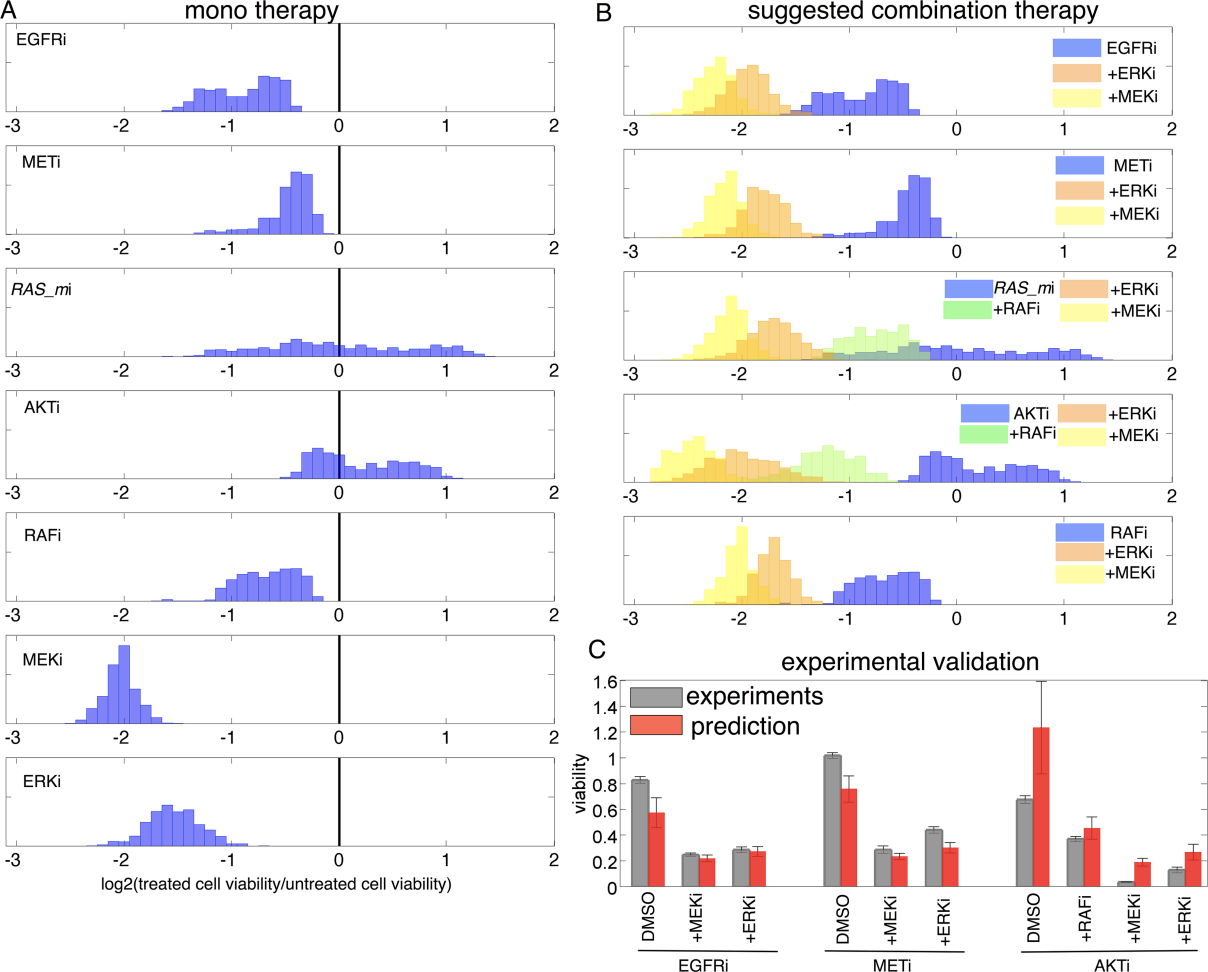
Model prediction and validation. (A) Histograms of relative cell viabilities (log2 scale) of all in silico cells after treatments of EGFRi, METi, *RAS_m*i, AKTi, RAFi, MEKi, and ERKi. First, a bimodal distribution EGFRi-treated in silico cells (one mode: −1.25; second mode: −0.5). Second, a skewed distribution after METi (a skew toward 0; 0: no change). Third, a uniform distribution in response to *RAS_m*i (almost uniform distribution from −1.5 to 1.5). Fourth, a slight bimodal distribution after AKTi. Fifth, a distributional response after RAFi. Sixth, a normal distribution in response to MEKi. Seventh, a normal distribution after ERKi. (B) Histograms of relative cell viabilities of all in silico cells in log2 scale. First: EGFRi only (blue), EGFRi/ERKi (orange), and EGFRi/MEKi (yellow). Second: METi only (blue), METi/ERKi (orange), and METi/MEKi (yellow). Third: *RAS_m*i only (blue), *RAS_m*i/RAFi (green), *RAS_m*i/ERKi (orange), and METi/MEKi (yellow). Fourth: AKTi only (blue), AKTi/RAFi (green), AKTi/ERKi (orange), and AKTi/MEKi (yellow). Fifth: RAFi only (blue), RAFi/ERKi (orange), and RAFi/MEKi (yellow). (C) Validation. Model predicted relative cell viabilities (red bars) and experimental data (gray bars) after 10 different treatments. First: EGFRi, EGFRi/MEKi, and EGFRi/ERKi. Second: METi, METi/MEKi, METi/ERKi. Third: AKTi, AKTi/RAFi, AKTi/MEKi, AKTi/ERKi. The numerical data used in Fig 3 are included in the second sheet S1 Data. AKT (PKB), protein kinase B; DMSO, Dimethyl sulfoxide (control); EGFR, epidermal growth factor receptor; ERK, extracellular receptor kinase; MEK, mitogen-activated protein kinase kinase; MET (c-MET), tyrosine-protein kinase Met or hepatocyte growth factor receptor (HGFR); RAF, rapidly accelerated fibrosarcoma; RAS, rat sarcoma.

We simulated responses of in silico cells to seven different inhibitors (EGFRi, METi, *RAS_m*i, AKTi, RAFi, MEKi, and ERKi). Of note, cell viability in untreated conditions is set to be a single constant value (cell viability_untreated_ ≡ 1). It is also worth noting that *RAS_m*i is assumed to inhibit only *RAS_m* (RAS mutant), not RAS_w. Distributions of relative cell viability (log2 scale, log_2_ (treated/untreated)) of all in silico cells are presented in Fig 3A. Similar to the experimental results (Fig 2B–2D), MEKi and ERKi reduced average cell viability significantly, whereas mean effects of EGFRi, METi, and AKTi are marginal. These results reveal quite heterogeneous responses to drugs, which could be assessed by experimental approaches [72, 73]. For example, the distribution of EGFRi treatment is bimodal due to a bimodal distribution of trained MEK-ERK weights (S1 Table and S2 Fig), suggesting the presence of a subpopulation that responds significantly differently to drug from the rest of the population (S2 Fig, green versus pink).

### In silico–predicted rational drug combinations

With our calibrated in silico cell lines, we next examine which drug combinations significantly reduce cell viability and which show marginal effects. For example, what should be cotargeted with AKTi to decrease cell viability significantly? The model predicts that activation of alternative pathways under therapy may provide an escape route to therapy. For example, AKT inhibitor induced increased activity of ERK and ribosomal S6 kinase (RSK) (S3 Fig). Cells with high ERK and RSK activity display resistance (cell viability >0) under the inhibitor, which implies that simultaneous inhibition of this alternative pathway would overcome resistance. We reasoned that combination targeting of proteins that are highly correlated with relative cell viability under a given treatment would be beneficial. In order to identify such protein nodes, scatter plots between predicted protein activity (phosphorylation) and predicted relative cell viability were considered (S4 Fig). Then, the Pearson’s coefficient was calculated for all cases. We observed that, under multiple treatment conditions—including inhibition of EGFR, MET, *RAS_m*, AKT, and RAF—ERK and RSK showed the highest correlation with relative cell viability (S4 Fig, pink box). This suggests that co-inhibition of ERK (by MEKi) or RSK (by ERKi) activity with other therapies would decrease cell viability most significantly. Further simulation revealed an additional application of either MEKi or ERKi to each—EGFRi, METi, *RAS_m*i, RAFi, and AKTi—significantly decreased cell viability compared to monotherapy of EGFRi, METi, *RAS_m*i, RAFi, and AKTi (Fig 3B). We tested some of these model predictions experimentally and validated, to some degree, the model’s predictive ability (Fig 3C).

### Hierarchical clustering of in silico cell responses

**Fig 4:**
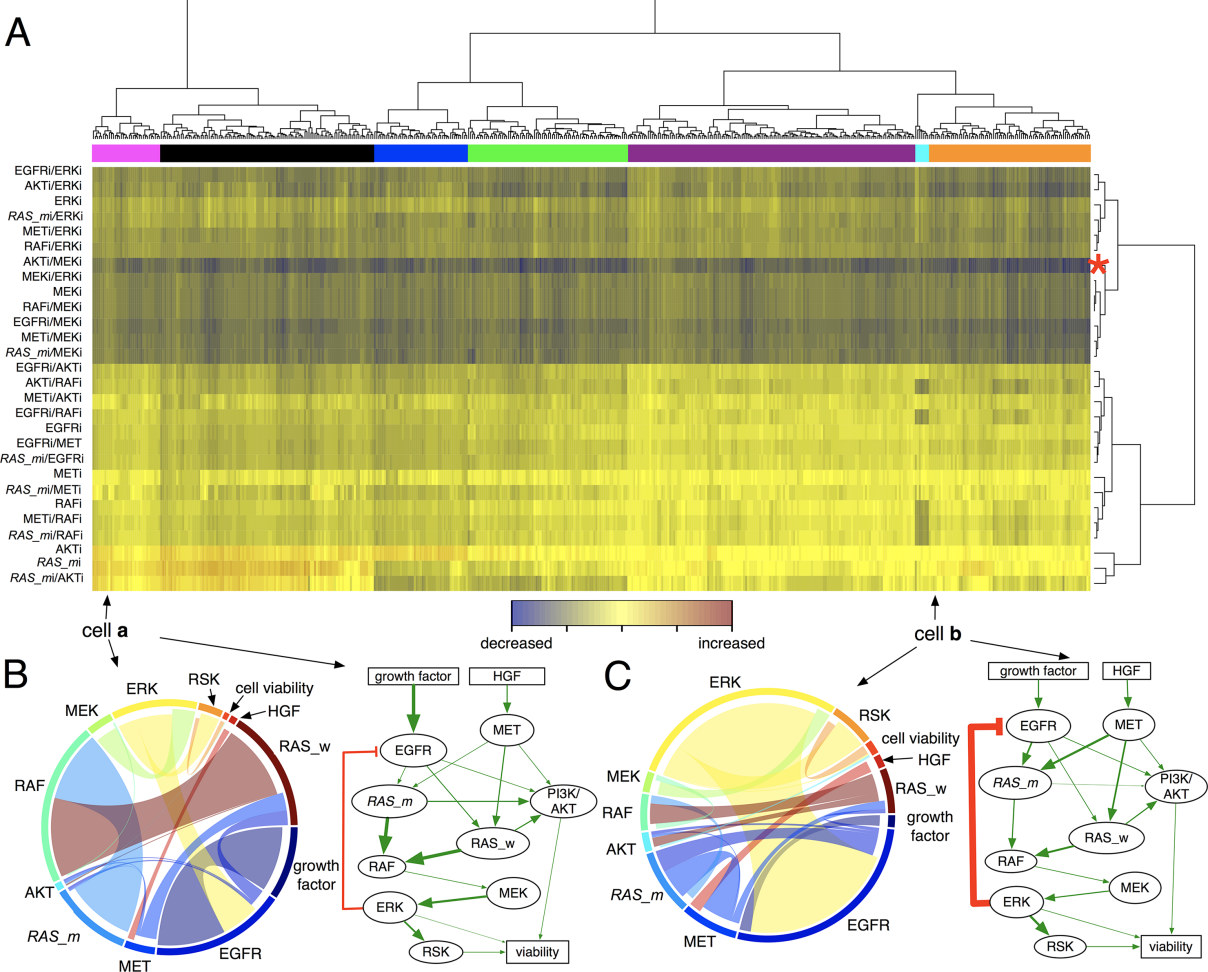
Model predicted combination therapy effect. (A) Hierarchical clustering and heat-map of relative cell viabilities after 28 different treatments. Simulated treatment responses (cell viability changes) were clustered using an unbiased hierarchical method with a Euclidian distance function, resulting in 7 different clusters (pink, black, blue, green, purple, cyan, and orange color) indicated by color bar on the top of heat-map. Each row indicates an individual therapy. Each column indicates an individual in silico cell. Blue to yellow bars: decrease to increase. The asterisk [*] indicates relative cell viability after a combination therapy of AKTi with MEKi. (B–C) Chord diagrams and weighted network diagrams of the representative in silico cells **a** and **b**. Chord diagram: each node in the circle represents each protein node in the network model, represented by different colors. The thickness of chord between two protein nodes represents a weight between two protein nodes (weight, *w*_*ij*_). The chords are directed, colored by originating sector color. For example, the interaction between *RAS_m* and RAF is depicted as a light blue chord because the direction is from *RAS_m* to RAF (*RAS_m* → RAF; color of *RAS_m* sector: light blue). Weighted network diagram: the width of each edge represents the weight. A thicker edge represents a larger weight between two proteins. The numerical data used in Fig 4 are included in the third sheet S1 Data. AKT (PKB), protein kinase B; EGFR, epidermal growth factor receptor; ERK, extracellular receptor kinase; HGF, hepatocyte growth factor; MEK, mitogen-activated protein kinase kinase; MET (c-MET), tyrosine-protein kinase Met or hepatocyte growth factor receptor (HGFR); PI3K, phosphoinositide 3-kinase; RAF, rapidly accelerated fibrosarcoma; RAS, rat sarcoma; RAS_m, mutated RAS; RAS_w, wild-type RAS; RSK, ribosomal S6 kinase.

We next systematically assessed cell viability reduction to all mono and combination therapies using an unsupervised hierarchical clustering approach to classify cell populations on the basis of their treatment response (relative cell viability). The treatments were categorized into multiple groups (Fig 4A, a tree diagram on the right end of heat-map). The combination of AKTi with MEKi is uniformly effective to all the cells (see the red asterisk [*] row, dark blue across all in silico cells, with little variation between cells). This combination (MEKi/AKTi) has previously been shown to be effective in NSCLC both in vitro and in vivo [74]. A striking variation is observed in response to the treatments of AKTi, *RAS_m*i, and *RAS_m*i/AKTi (Fig 4A, red bars in the first two groups versus dark blue to yellow bars in the rest of the groups). The first two clusters (pink and black color on the top of the heat-map) are associated with poor responses (little to no reduction of cell viability after a given therapy), while others are correlated with good treatment outcomes (significant reduction of cell viability after a given therapy).

### Possible mechanisms of response and resistance

Why are some cells sensitive to a given therapy while others are resistant to the same therapy? We hypothesize that this differential drug sensitivity, at least within the context of our model, must be attributed to differential protein activity as modulated by protein–protein interactions (i.e., the weights). To highlight possible mechanisms, we visualized the weights between protein nodes (*W*_*ij*_ in our model) using both circular chord diagrams [75] and network diagrams with weighted edges (Fig 4, bottom panels; left: chord diagram; right: weighted network with different edge widths). In the circular diagrams, protein nodes are arranged around a circle with the weight between protein nodes connected to each other through the use of arcs. The width of each arc is determined proportionally by the weight between two protein nodes. To illustrate differences in signaling, we selected two representative in silico cells (Fig 4A and 4B), where cell a is resistant to AKTi, *RAS_m*i, and *RAS_m*i/AKTi and cell b is more sensitive to these therapies. In addition, we compared ranges of all weights of all in silico cells between clusters defined by the hierarchical clustering (S5A Fig). We observe heterogeneity of weights within each cluster and between clusters. Differences between clusters are significant for some weights such as weights of growth factor EGFR, EGFR-RAS_w, MET-RAS_w, RAS_w-RAF, and MEK-ERK (S5A Fig).

### Heterogeneous responses to HGF stimulation

**Fig 5:**
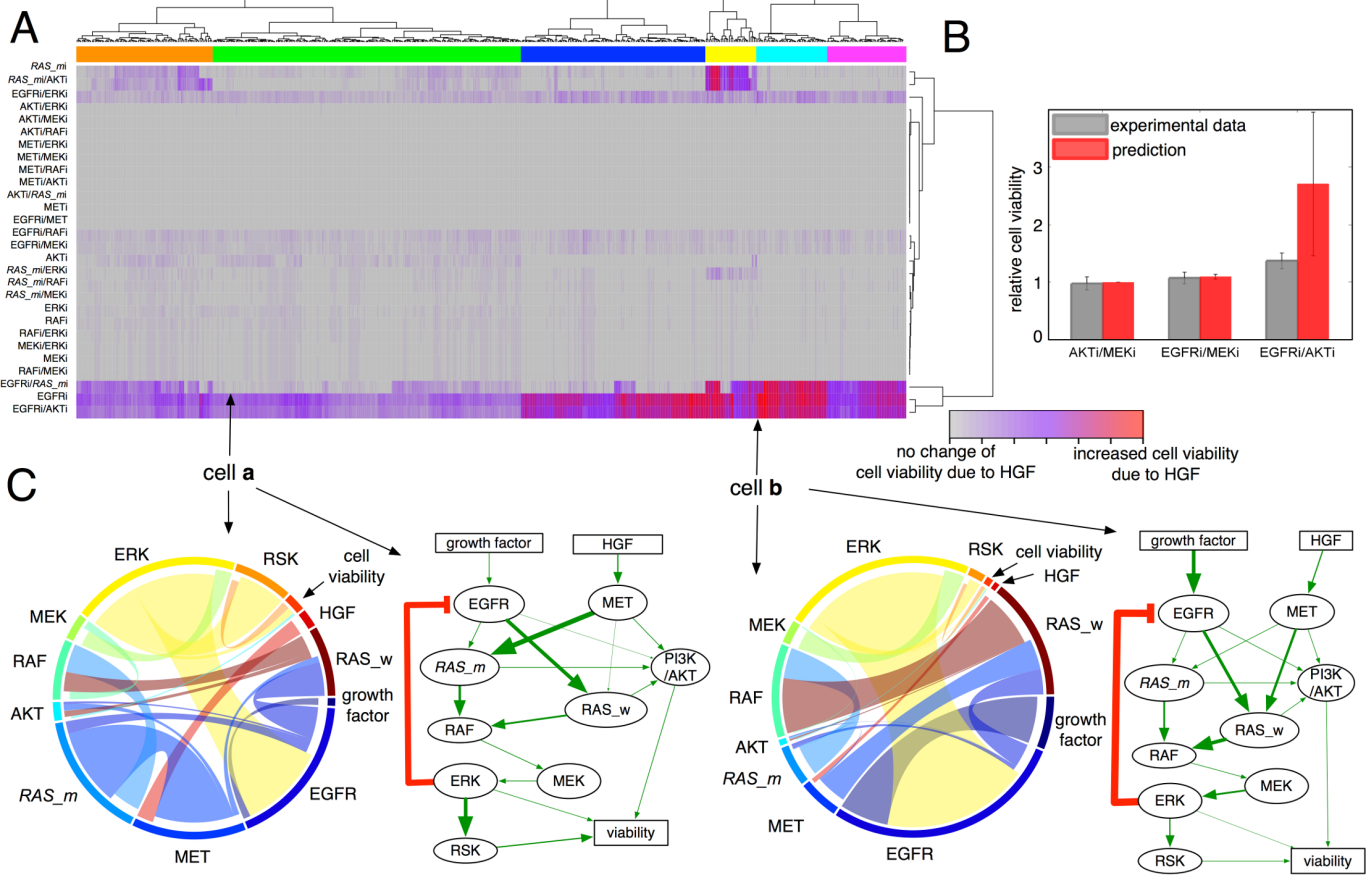
Effect of HGF stimulation on treatment responses. (A) Heat-map of cell viability changes due to HGF stimulation (log_2_ (treated with HGF/untreated)-log_2_ (treated without HGF/untreated)). An unbiased hierarchical clustering separated the cell population into 6 different clusters (indicated by colors on the top of heat-map). Each row indicates a treatment, and each column indicates an in silico cell. Gray to red: no change to increase due to HGF. (B) Model validation. Comparisons between experimental data (gray bars) and model predictions (red bars) after three different combinations (AKTi/MEKi, EGFRi /MEKi, and EGFRi/AKTi). Relative change of cell viability in treated-with-HGF condition to one in treated-without-HGF condition (no HGF) is reported. (C) Chord diagrams and weighted network diagrams that visualize weights between two protein nodes in the representative in silico cell **a** and **b**. The numerical data used in Fig 5 are included in the fourth sheet S1 Data. AKT (PKB), protein kinase B; EGFR, epidermal growth factor receptor; ERK, extracellular receptor kinase; HGF, hepatocyte growth factor; MEK, mitogen-activated protein kinase kinase; MET (c-MET), tyrosine-protein kinase Met or hepatocyte growth factor receptor (HGFR); PI3K, phosphoinositide 3-kinase; RAF, rapidly accelerated fibrosarcoma; RAS, rat sarcoma; RAS_m, mutated RAS; RAS_w, wild-type RAS; RSK, ribosomal S6 kinase.

We next asked how an additional microenvironmental stimulation would modulate the responses to targeted treatments. To address this question, all in silico cells were treated in the presence of HGF, a significant stromal factor that contributes to drug resistance [35, 36]. An unsupervised hierarchical clustering on the basis of cell viability changes, from the no-HGF condition, separated the treatments into several groups (Fig 5A, clustering of treatments on the right tree diagram). The analysis also classified the in silico cells into several groups based on cell viability changes due to HGF stimulation (Fig 5A, tree diagram on the top of heat-map).

To test some of these model predictions, we considered three different combination therapies, AKTi/MEKi, EGFRi/MEKi, and EGFRi/AKTi. Of note, the model predicted that the treatments AKTi/MEKi and EGFRi/MEKi are not affected by HGF stimulation (Fig 5B, first two red bar graphs). The experimental data matched well with these predictions (Fig 5B, first two gray bar graphs). The model also predicted that the effect of combination therapy EGFRi/AKTi is significantly modulated by HGF stimulation (Fig 5B, the third red bar). This was also corroborated by experiment (Fig 5B, the third gray bar).

### Possible mechanisms explaining HGF modulation

The suite of in silico cells is differentially affected by HGF stimulation. For example, some cells are not affected by the HGF stimulation (e.g., gray bars in EGFRi/*RAS_m*i in Fig 5A), while others are significantly affected by stimulation (e.g., red bars in EGFRi/*RAS_m*i in Fig 5A). Why are some cells affected by HGF stimulation, while others are not? To understand why this is the case, we selected two representative cells (**a** and **b**) and visualized the weights between protein nodes using both chord diagrams and weighted network diagrams (Fig 5C, bottom panels). In cell **a**, the influence of MET on RAS_w is relatively small (Fig 5C, thin blue chords from MET → RAS_w). In contrast to cell **a**, the influence of MET on RAS_w in cell **b** is much stronger (Fig 5C, cell **b**, thick blue chord from MET → RAS_w). This may explain why cell **b** increased its viability significantly upon HGF stimulation compared to no-HGF condition. In addition, distributions of all weights in each cluster show differential activity of proteins and its effects across all six different clusters (S5B Fig).

### Competition between in silico cells drives different dominant populations

**Fig 6:**
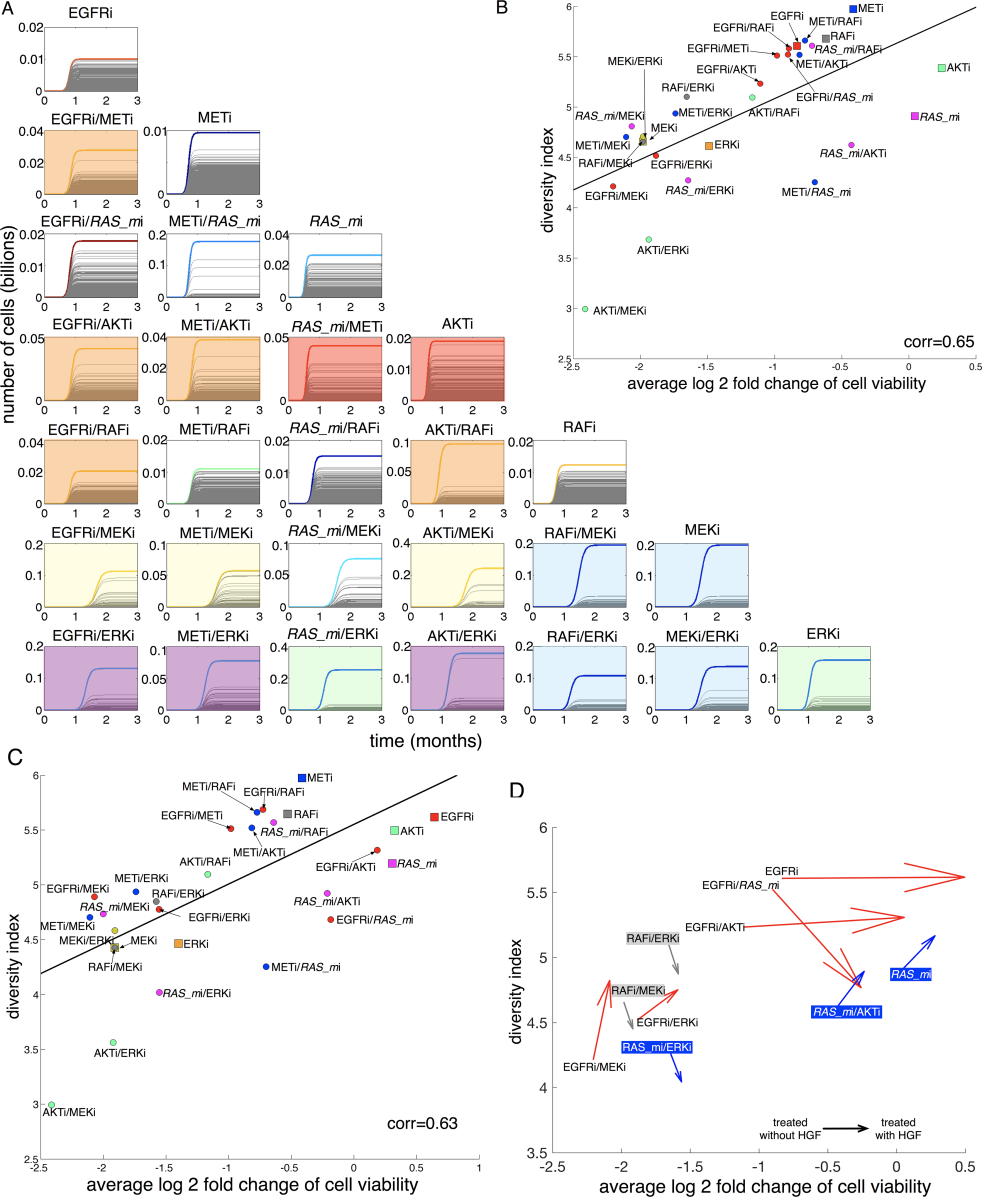
Effect of homogeneous, resource-limited microenvironment on treatment outcomes and heterogeneity. (A) The growth dynamics of all in silico cells treated with various therapies for three months in a homogeneous and resource-limited microenvironmental condition. A color line at each treatment indicates the most dominant cell after a given therapy. Color-shaded boxes indicate treatments selecting for the same dominant in silico cell. Orange box: dominant cell (cell ID: 342) after treatments of EGFRi/METi, EGFRi/AKTi, METi/AKTi, EGFRi/RAFi, and AKTi/RAFi. Red box: dominant cell (ID: 417) after *RAS_m*i/METi and AKTi. Yellow box: dominant cell (ID: 337) after EGFRi/MEKi, METi/MEKi, and AKTi/MEKi. Blue box: dominant cell (ID: 36) after RAFi/MEKi, MEKi, RAFi/ERKi, and MEKi/ERKi. Purple box: dominant cell (ID: 156) after EGFRi/ERKi, METi/ERKi, and AKTi/ERKi. Green box: dominant cell (ID: 113) after ERKi and ERKi/*RAS_mi*. (B) Linear relationship between average relative cell viability (log2 scale) and post-treatment Shannon indexes. Linear correlation constant: 0.65. Square: monotherapy; circle: combination therapy. Red: EGFRi alone or combination with METi, *RAS_mi*, AKTi, RAFi, MEKi, or ERKi. Blue: METi alone or combination with *RAS_m*i, AKTi, RAFi, MEKi, or ERKi. Pink: *RAS_m*i alone or combination with RAFi, AKTi, MEKi, or ERKi. Green: AKTi alone or combination with RAFi, MEKi, or ERKi. Gray: RAFi, RAFi/MEKi, RAFi/ERKi. Yellow: MEKi, MEKi/ERKi. Orange: ERKi. (C) Linear relationship between average relative cell viability (log2 scale) and post-treatment (with HGF stimulation) Shannon indexes. Linear correlation constant: 0.63. Square: monotherapy; circle: combination therapy. Color definitions are the same as panel B. (D) Therapies affected by HGF significantly. Each arrow starts from a point without HGF stimulation to a point with HGF stimulation. Gray: RAFi/ERKi and RAFi/MEKi. Blue: *RAS_m*i, *RAS_m*i/AKTi, *RAS_m*i/ERKi. Red: EGFRi, EGFRi/*RAS_m*i, EGFRi/AKTi, EGFRi/MEKi, and EGFRi/ERKi. The numerical data used in Fig 6 are included in the fifth sheet S1 Data. AKT (PKB), protein kinase B; corr, linear correlation; EGFR, epidermal growth factor receptor; ERK, extracellular receptor kinase; HGF, hepatocyte growth factor; MEK, mitogen-activated protein kinase kinase; MET (c-MET), tyrosine-protein kinase Met or hepatocyte growth factor receptor (HGFR); PI3K, phosphoinositide 3-kinase; RAF, rapidly accelerated fibrosarcoma; RAS, rat sarcoma; RAS_m, mutated RAS; RAS_w, wild-type RAS.

So far, treatment responses were assessed as if all in silico cells were treated in isolation. To address the effects of cell competition on treatment outcomes, we simulated all cells growing together under treatment with various inhibitors in a homogeneous, resource-limited microenvironment (Methods section). The entire in silico cell population responds in a similar way on some therapies (Fig 6, e.g., EGFRi, METi, RAFi, EGFRi/*RAS_m*i, etc.), but under other therapies (Fig 6, e.g., MEKi, ERKi, MEKi/ERKi, AKTi/RAFi, etc.)—due to differential viability—some cells became dominant. Interestingly, some treatments simultaneously selected for the same dominant in silico cell (Fig 6, color-shaded boxes). Addition of HGF to some treatments (e.g., EGFRi, *RAS_m*i, RAFi) significantly changes the fitness of in silico cells and thus drives a different dominant in silico cell (S6 Fig, dotted lines in no-HGF vs solid lines in HGF).

### Assessment of post-treatment in silico cell population heterogeneity

We assessed post-treatment population heterogeneity by measuring the Shannon index (*H*(*x*) = −∑_*i*_*p*_*i*_(*x*)log(*p*_*i*_(*x*)), where *p*_*i*_(*x*) is the probability of finding an in silico cell *i* after a given therapy). We compared the index with average cell viability change (Fig 6B). The two are linearly correlated (*ρ* = 0.65), implying that the less effective a treatment is in controlling the average in silico population growth, the more heterogeneous the post-treatment population would be (Fig 6B). Combination therapies not only display a better average treatment response but also a less diverse post-treatment population (Fig 6B, boxes versus circles). Among all combination therapies, those that combined either with ERKi or MEKi display much better average therapeutic responses (Fig 6B, small cell viability). These treatments not only effectively decrease average cell viability but also lead to a less diverse post-treatment population (e.g., Fig 6B, EGFRi/[ERKi or MEKi] vs EGFRi/[METi,AKTi,RAFI] in red-color circles). HGF stimulation minimally affected the linear relationship between post-treatment heterogeneity and average cell viability reduction (Fig 6C, *ρ* = 0.63 vs Fig 6B, *ρ* = 0.65). However, a few treatments did elicit significant changes in both average response and heterogeneity due to HGF stimulation (Fig 6D).

### Impact of microenvironmental heterogeneity on targeted therapy outcomes

**Fig 7:**
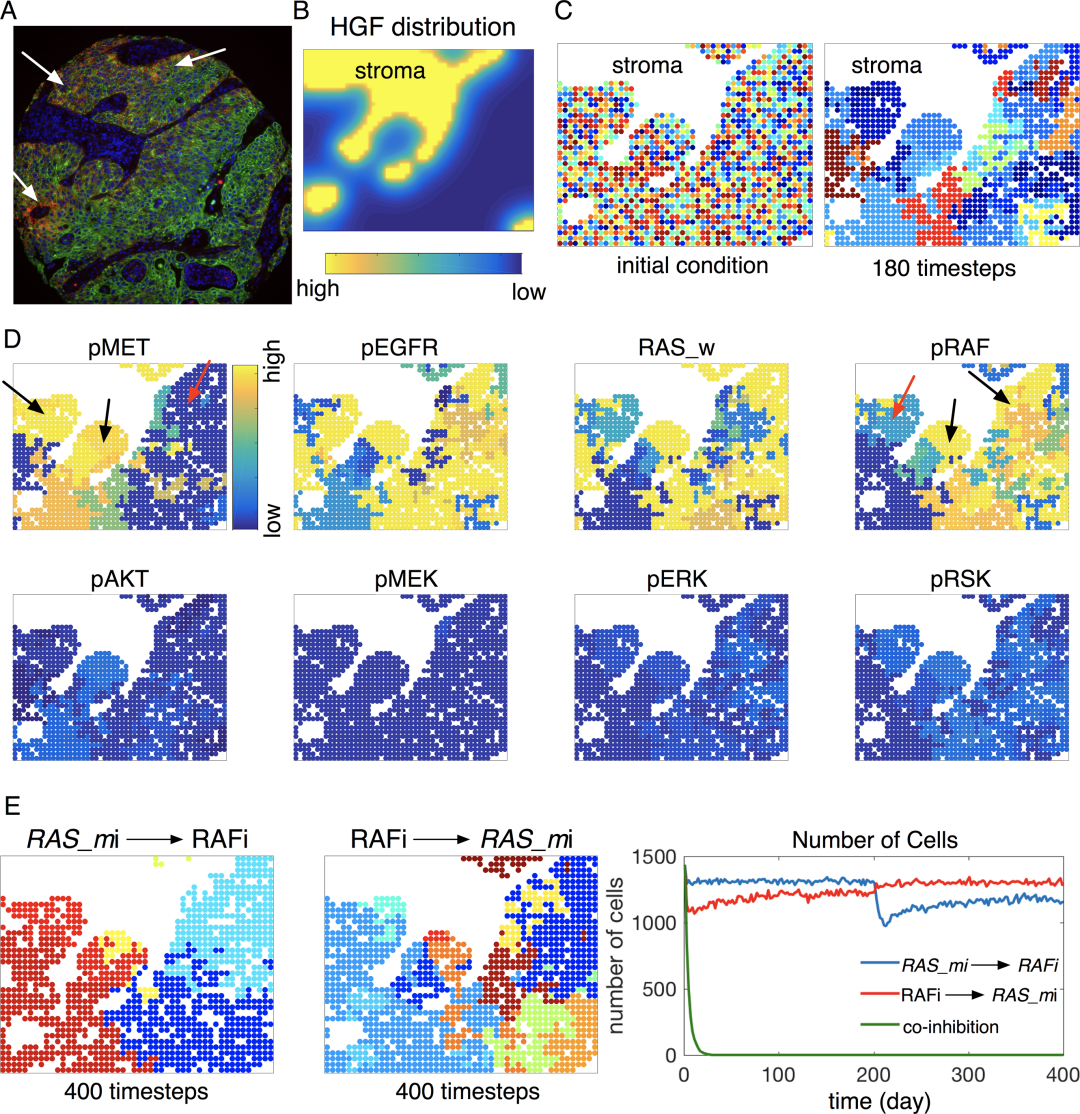
Effect of microenvironmental heterogeneity on a targeted therapy outcome and combination therapy. (A) Heterogeneous EGFR activity in a lung squamous cell carcinoma. Representative image of red foci showing EGFR:GRB2 proximity. Tumor cells (green) are stained with a cytokeratin antibody demarcating epithelial origin. Nuclei (blue) are stained with DAPI. Image was acquired at 200x. (B) HGF distribution. The Eq (3) was solved assuming the following parameters: *γ* = 1.0, diffusion rate *D* = 0.04, and decay rate *λ* = 0.001. To be consistent with the choice of parameter in the pathway model (Eq [1]), we use a scaling factor *ω* (*ω* ≡ 10) (i.e., HGF input in the pathway model *x*_2_ = *ωH*(*x*,*t*), *H*(*x*,*t*): solution obtained using the assumed parameters). (C) First, an initial randomized configuration of cells (domain size: 50 cells × 38 cells). Color represents different in silico cells. Second, a snapshot of *RAS_m* inhibitor simulation (day 180). (D) Simulated protein activity after 180 days of *RAS_m* inhibition. The activities of MET, EGFR, RAS_w, and RAF are heterogeneous (yellow to blue color), while those of AKT, MEK, ERK, and RSK are less heterogeneous (blue color). (E) Comparison of combination therapies for 400 days. First, a snapshot of simulation (day 400) after a sequential therapy of *RAS_m*i for the first 200 days and then RAFi for the rest 200 days (200 days of *RAS_m*i → 200 days of RAFi). Color represents different in silico cells. Second, a snapshot of simulation (day 400) after a sequential therapy of RAFi for the first 200 days, then *RAS_m*i for the remaining 200 days (200 days of RAFi → 200 days of *RAS_m*i). Third: the number of cells over time during the two sequential therapies of *RAS_m*i and RAFi (blue: *RAS_m*i → RAFi, red: RAFi → *RAS_m*i) and a concurrent therapy of the two (green: *RAS_m*i/RAFi). The numerical data used in Fig 7 are included in the sixth sheet S1 Data. AKT (PKB), protein kinase B; DAPI, 4',6-diamidino-2-phenylindole; EGFR, epidermal growth factor receptor; ERK, extracellular receptor kinase; GRB2, growth factor receptor bound protein 2; HGF, hepatocycte growth factor; MEK, mitogen-activated protein kinase kinase; MET (c-MET), tyrosine-protein kinase Met or hepatocyte growth factor receptor (HGFR); RAF, rapidly accelerated fibrosarcoma; RAS, rat sarcoma; RSK, ribosomal S6 kinase.

Because activated receptors require multiple protein interactions to activate downstream signaling, we have utilized proximity ligation assays that measure the functional association of RTKs and adaptor proteins. Using NSCLC patient specimens and xenograft models, we have previously identified an association of EGFR:GRB2 complexes and response to EGFR inhibition [76] and more recently identified a correlation between MET:GRB2 complexes and response to MET kinase inhibitors [77]. Using this approach, we consistently observe spatial heterogeneity in abundance of RTKs binding to adaptor complexes. The abundance is often highest at tumor regions that are adjacent to stromal regions (Fig 7A).

Motivated by this experimental observation (Fig 7A), we developed an HCA model to investigate the effects of microenvironmental heterogeneity on treatment outcomes (See Methods section and S1 Fig). A steady state configuration of HGF is considered throughout the whole simulation (See Methods section and Fig 7B). We randomly initialized in silico cells that contain calibrated signaling networks (Fig 2) in the domain to mimic a slice of tumor tissue (Fig 7C first, time step = 0; domain size: 50 cells × 38 cells). As proof of concept, we simulated *RAS_m* inhibition. After 180 days of the inhibition, distinct cells emerge near stroma (high HGF) in contrast to the nonstroma region (Fig 7C second, light- and dark-blue cells near stroma versus orange, red cells elsewhere). A clearer separation among the cell population emerges as therapy continues (S1 Movie). We also observed heterogeneous protein activity across the tissue (Fig 7D and S2 Movie). The signaling responses of some cells are affected by HGF (Fig 7D, e.g., high MET and RAF, black arrows near stroma), while signaling in other cells is not (Fig 7D, e.g., low activity of MET and RAF near stroma, red arrow).

The treatment selects for cells with high MET and RAF activity (phosphorylation), especially residing near the stroma (high HGF), which suggests that a therapy of METi or RAFi in addition to *RAS_m*i may be more effective. To test this suggestion, we simulated two sequential therapies of *RAS_m*i and RAFi and one concurrent therapy (Fig 7E and S3–S6 Movies). Depending on the order of the sequence (*RAS_m*i first versus RAFi first), different patterns of cells emerge after 400 days of treatment (Fig 7E, first versus second). Importantly, a concurrent combination of the two inhibitors was effective enough to eradicate all cells in this small region just after 30 days of treatment (S5 Movie and Fig 7E third, number of cells). The direct spatial competition of each cell within the tissue directly facilitated this result. To be more relevant to the clinical timeframe, we also simulated a shorter treatment schedule (e.g., 60 days) and observed similar cell behavior (S7 Fig).

Until now, we assumed that the initial states of the protein activities in our in silico cells were all zero. In order to examine the impact of changing this on the above treatment outcomes, we randomly seeded in silico cells in a slice of tissue (Fig 7C), and for each in silico cell, a random number was assigned to each initial protein activity. Then, we simulated *RAS_m* inhibitor for 30 days using our HCA model (S1 Fig) and repeated this process 100 times. The resulting configurations display some degree of heterogeneity (S8A Fig, shows representative results for three different initial configurations) due to cell–cell spatial competition as well as variable HGF modulation of the in silico cells. The HGF selects for cells whose cell viability is significantly modulated by HGF (violet to red cells near stroma). To illustrate this more accurately, we quantified the total number of surviving cells at time step 30 for each simulation (see S8B Fig for distributions) and classified them in terms of HGF modulation (S8C Fig). The treatment consistently selected for certain cells (S8C Fig), some of which are more influenced by HGF than others (S8C Fig).

## Discussion

We implemented an integrated mathematical and experimental approach to develop a panel of in silico cells that readily reproduced average kinase inhibitor responses in two different microenvironments (HGF and no-HGF). The mathematical model of a simplified oncogenic *RAS*-driven MAPK and AKT-PI3K pathway describes weighted interactions between proteins in the pathway. The weights here may represent relative protein abundance or protein activity. The calibrated model predicted heterogeneous responses to kinase inhibitors due to differential activities of proteins from the in silico cells under a given therapy condition. In addition, the model identified a combination therapy that effectively reduced cell viability across the entire in silico cell population (Fig 4, AKTi/MEKi). Critically, the effects are not modulated by HGF stimulation (Fig 5). This combination has been shown to be effective in NSCLC both in vitro and in vivo [74]. Integrating the pathway model into a two-dimensional lattice-based model allowed us to take a significant step toward modeling the multiscale behavior of cancer by bridging the signaling, cell, and multicellular scales with feedback from the microenvironment. We were also able to simulate the impact of an inhibitor on a tissue structure composed of tumor and stroma, showing complex signaling responses and selection for distinct in silico cells by the stroma (Fig 7 and S1 and S2 Movies), which highlighted a novel combination therapy (Fig 7 and S3–S6 Movies).

We are only just beginning to understand the importance of nongenetic heterogeneity [19, 78]. Much more needs to be done in teasing apart the different scales of heterogeneity (genetic, cellular, microenvironmental), how they interact and modulate one another, and how this might alter our current combination treatment strategies. Variable protein activity has been observed in previous studies of isogenic cancer cell lines, revealing that single-cell heterogeneity and protein–protein interaction strength is different [73]. A more recent study, of an isogenic cancer, showed striking variation in genetic, cellular, and phenotypic heterogeneity [79]. Our own experiments and simulations showed heterogeneous cancer cell signaling in a section of lung adenocarcinoma (Fig 7) [76, 77].

There are other modeling approaches for analysis of signaling pathways including logical, Boolean, and artificial neural network (reviewed in [37]). In particular, several recent studies developed integrated approaches of mathematical modeling with systematic perturbation experiments applying various kinase inhibitors to cancer cells. Some of these studies proposed novel combination therapies [43, 51, 54], just as we have. However, in addition to predicting average cell viability, we also consider post-treatment heterogeneity and, critically, the impact of the microenvironment. Historically, Boolean models have been used in understanding cancer cell–signaling responses. To investigate applicability of such a Boolean approach, we constructed an equivalent Boolean model of the signaling pathway (S2 Text and S9 Fig). This model predicted the treatment combinations (AKTi/MEKi and AKTi/RAFi) consistent with experimental data (Fig 3C, two out of seven different combination therapies). However, the other five combination therapies were not consistent with experimental data (yellow asterisks in S9C Fig). Taken together, these results suggest that the Boolean model (S9A Fig) is insufficient to predict combination therapies.

To obtain a tractable model, several simplifications have been made in this study. The model considered only two microenvironmental variables (HGF and a generic growth factor) on a two-dimensional square lattice, where the heterogeneous population of in silico cells mimicked a slice of lung tissue. Although three-dimensional models will describe these dynamics in a more realistic way, the key predictions—for example, the effect of stroma—will be consistent in a three-dimensional setting. Other simplifying aspects of the HCA approach included not considering tissue mechanical properties as well as constraining cell movement to orthogonal neighbors with discontinuous displacement. A potential alternative approach would be to consider off-lattice models that allow force-driven interactions that better describe mechanical aspects of tumor growth [80–83] and tumor morphology [84–86]. We are also acutely aware that the signaling network considered is only a fragment of a much larger and far more complex signaling cascade that turns external signals into phenotypic decisions. Because not all proteins in cancer cells are directly measurable due to experimental limitations, many players and intermediate proteins in the pathway are not included in our study. Therefore, an interaction between two proteins represents diverse influence of one entity on other entity in steady state, such that an entity can be a microenvironmental variable, an intracellular protein, or cell viability. We emphasize that, by definition, all models are but simplifications of reality and the true utility of a model is not that it can mimic reality but that it provides useful insight into the system. Despite this simplicity, our integrated approach provided multiple testable hypotheses for the complex *KRAS* NSCLC cell signaling network, proposed possible drug resistance mechanisms, and suggested better treatment strategies.

Finally, while we relied on western blots for protein activity (phosphorylation) readouts, alternative approaches such as Reverse Phase Protein Array exist [87]. The important step, however, was combining this average protein activity with prior information about network connectivity, allowing us to generate a suite of in silico cell lines that not only reproduced this average behavior but also gave insights into potential single-cell variability. This highlights a key need to improve our understanding of heterogeneous cell signaling networks: single-cell profiling. Such data would ideally include intracellular, cellular, and phenotypic profiling in multiple, uniform microenvironments. However, our results also emphasize the importance of interactions between heterogeneous cell populations and spatially structured environments. Therefore, while quantifying single-cell dynamics will provide key information about intrinsic cell heterogeneity, to fully understand how these differences impact treatment responses, we must consider how interactions that change through space and time alter this heterogeneity and thus treatment outcomes.

## Supporting information

S1 Fig Flowchart of each cell in the HCA model. Each cell contains its own signaling network (calibrated network model, Fig 2A) and processes signaling to determine its viability. If the viability is low, the cell commits death with a probability of the viability. Otherwise, the cell waits for the next time step. If the cell viability is not too low, we check for an empty space in its nearest four neighbors (north, east, south, west from the cell). If there is an empty space, the cell divides with a probability of the viability. If there is no empty space, the cell becomes quiescent and waits for next time step. HCA, hybrid cellular automata.
(TIFF)

S2 Fig The in silico cells were divided into two groups based on MEK-ERK weights (large weights: Green versus small weights: Pink; a distribution figure on the right upper corner). Then, distributions of treatment responses for each group were considered. The in silico cells with larger MEK-ERK weights seem to be more sensitive to both EGFRi and RAFi and are more resistant to AKTi than the ones with smaller MEK-ERK weights. AKT (PKB), protein kinase B; EGFR, epidermal growth factor receptor; ERK, extracellular receptor kinase; MEK, mitogen-activated protein kinase kinase; RAF, rapidly accelerated fibrosarcoma.
(TIFF)

S3 Fig Activation of alternative pathway in response to AKTi. A scatter plot of relative cell viability (ratio of cell viability after treatment to cell viability before treatment, log 2 scale) as a function of relative activities of ERK and RSK (ratio of protein activity after treatment to protein activity before treatment, log 2 scale) is given. Color represents cell viability (blue: small and yellow: large). Gray plane indicates no change of cell viability. AKT (PKB), protein kinase B; ERK, extracellular receptor kinase; RSK, ribosomal S6 kinase.
(TIFF)

S4 Fig Linear correlation between cell viability and each protein activity. Scatter plots of cell viability changes (*y* axis) against individual protein activity changed (labeled on the bottom, EGFR, MET, *RAS_m*, RAS_w, AKT, RAF, MEK, ERK, RSK) after each monotherapy (labeled on the top of each row; first row: EGFRi; second row: METi; third: *RAS_m*i; fourth: AKTi; fifth: RAFi; sixth: MEKi; seventh: ERKi). Each dot represents an individual in silico cell. Dotted lines: no change; colored box: highly correlated relations; r: correlation coefficient. AKT (PKB), protein kinase B; EGFR, epidermal growth factor receptor; ERK, extracellular receptor kinase; MEK, mitogen-activated protein kinase kinase; MET (c-MET), tyrosine-protein kinase Met or hepatocyte growth factor receptor (HGFR); RAF, rapidly accelerated fibrosarcoma; RAS, rat sarcoma.
(TIFF)

S5 Fig Distributions of weights. (A) Box plots of weights from each cluster in Fig 4A. Colors correspond to the different clusters in Fig 4A. (B) Boxplots of weights from each cluster in Fig 5A. Colors correspond to the different clusters in Fig 5A.
(TIFF)

S6 Fig Each therapy was applied to all in silico cells with HGF stimulation for three months. Color-shaded boxes indicate treatments selecting the same dominant clone. Compared with no-HGF treatment (Fig 6A), the treatments of EGFRi/MEKi, *RAS_m*i/MEKi, and EGFRi/ERKi select a different dominant cell (dashed-color line vs solid-color line). AKT (PKB), protein kinase B; corr, linear correlation; EGFR, epidermal growth factor receptor; ERK, extracellular receptor kinase; HGF, hepatocyte growth factor; MEK, mitogen-activated protein kinase kinase; MET (c-MET), tyrosine-protein kinase Met or hepatocyte growth factor receptor (HGFR); PI3K, phosphoinositide 3-kinase; RAF, rapidly accelerated fibrosarcoma; RAS, rat sarcoma.
(TIFF)

S7 Fig Treatments considered in Fig 7 (*RAS_m*i → RAFi and RAFi → *RAS_mi*) were simulated over 60 days. (A) Cell configuration after 30 days of *RAS_m*i first followed by 30 days of RAFi. Different cells are represented by different colors. (B) Cell configuration after 30 days of RAFi first followed by 30 days of *RAS_m*i. Different cells are represented by different colors. (C) Total number of cells over time; blue: *RAS_m*i → RAFi; red: RAFi → *RAS_m*i. HCA, hybrid cellular automata; RAF, rapidly accelerated fibrosarcoma; RAS, rat sarcoma.
(TIFF)

S8 Fig We considered 100 different randomly selected initial conditions of protein states and simulated *RAS_m* inhibitor for 30 days. (A) Three different representative configurations of cells at time step 30. Color: HGF modulation, gray: no change of cell viability due to HGF stimulation; violet: significant increase of cell viability due to HGF. (B) Distribution of number of cells at time step 30. Boxplots of number of all of 500 cells at time step 30. “+” indicates outliers. The maximum difference of interquartile range is 30 cells (approximately 0.02% of total cell population). (C) Boxplots of the number of cells whose median cell numbers at time step 30 is greater than 0. Color: HGF modulation; gray: no change of cell viability due to HGF stimulation; violet: significant increase of cell viability due to HGF. HCA, hybrid cellular automata; HGF, hepatocyte growth factor; RAS, rat sarcoma.
(TIFF)

S9 Fig (A) Boolean network model. (B) Seven different attractors represented by different colors. (C) Active or inactive state of proteins and cell viability after various mono and combination therapies were applied. Yellow star: cell viability state that is inconsistent with experimental data (Figs 2 and 3).
(TIFF)

S1 Text RMSE formula used in Fig 2. RMSE, root-mean-squared-error.
(DOCX)

S2 Text Boolean network model. We constructed an equivalent Boolean model of the signaling pathway (S9 Fig). In the model, each node has a binary value (1: active, on; 0: inactive, off). The behavior of each node is modeled as a sequence of discrete steps in a Boolean function defining the value of a node on the next step based on values of its neighbor nodes (S9A Fig). For all nodes except EGFR, a node will be active if at least one of its neighbors is active. The node EGFR will be active if either growth factor is active or ERK is inactive (inhibitory regulation of EGFR by ERK). Of note, *RAS_m* node is always active, representing RAS mutation in the cell line (A549) used in our experiments. Assuming both of the input nodes—(growth factor and HGF) and *RAS_m*—are always active, all possible initial states (2^10^) are exhaustively simulated, using the R package *BoolNet* [88], until reaching attractors (steady states). The simulations converged on seven different attractors (S9B Fig). We then simulated seven different combination therapies that we tested in our experiments (Fig 3C). To simulate drug-induced inhibition, we made each target node constitutively inactive (e.g., EGFR = 0 for EGFRi; MET = 0 for METi; AKT = 0 for AKTi; ERK = 0 for MEKi; and RSK = 0 for ERKi). Two drug combinations result in an inactive viability state (S9C Fig, viability in red, AKTi/RAFi, AKTi/MEKi), which are consistent with both our modeling and experimental data (Fig 3C and Fig 4A). The Boolean network model predicts that other combinations are not effective (S9C Fig, active viability state in green), which are not consistent with both our model predictions and experimental data (yellow asterisks in S9C Fig vs Fig 3C). Taken together, these results suggest that this simple Boolean network is insufficient to recapitulate our experimental data.
(DOCX)

S1 Table For each weight of interaction, kernel density of distribution was estimated using R (gray probability density plots on the edge). Shannon index (SI) was also reported. A blue box indicates weights with the lowest Shannon index, while red boxes indicate weights with large Shannon index (>4.0).
(TIFF)

S1 Data Numerical data used in figures. (XLSX)

S1 Movie RAS_*m* inhibitor simulation (cells). (AVI)

S2 Movie RAS_*m* inhibitor simulation; protein phosphorylation (activity). Yellow: high activity; blue: low activity.
(AVI)

S3 Movie A sequential therapy of *RAS_m* inhibitor for the first 200 days and then RAF inhibitor for 200 more days. (AVI)

S4 Movie A sequential therapy of RAF inhibitor for the first 200 days and then *RAS_m* inhibitor for 200 days. (AVI)

S5 Movie Concurrent combination of *RAS_m* inhibitor with RAF inhibitor for 30 days. (AVI)

S6 Movie Temporal evolution of the number of cells (bar graphs; color: Different in silico cells). (AVI)

## Abbreviations

AKT (PKB): protein kinase B
DMSO: Dimethyl sulfoxide (control)
EGFR: epidermal growth factor receptor
ERK: extracellular receptor kinase
FDA: Food and Drug Administration
GRB2: growth factor receptor bound protein 2
HCA: hybrid cellular automata
HGF: hepatocyte growth factor
KRAS: Kirsten rate sarcoma
MAPK: mitogen-activated protein kinase
MEK: Mitogen-activated protein kinase kinase
MET (c-MET): tyrosine-protein kinase Met or hepatocyte growth factor receptor (HGFR)
NSCLC: non–small-cell lung cancer
ODE: ordinary differential equation
PI3K: phosphoinositide 3-kinase
RAF: rapidly accelerated fibrosarcoma
RAS: rat sarcoma
RMSE: root-mean-squared-error
RSK: ribosomal S6 kinase
RTK: receptor tyrosine kinase
Ser: serine
Thr: threonine
Tyr: tyrosine
