# Supplementary figures and images for: Cell signaling heterogeneity is modulated by both cell-intrinsic and -extrinsic mechanisms: An integrated approach to understanding targeted therapy
Source: PLoS Biol. 2018 Mar 9;16(3):e2002930. doi: 10.1371/journal.pbio.2002930 (PMC5844524; doi:10.1371/journal.pbio.2002930)

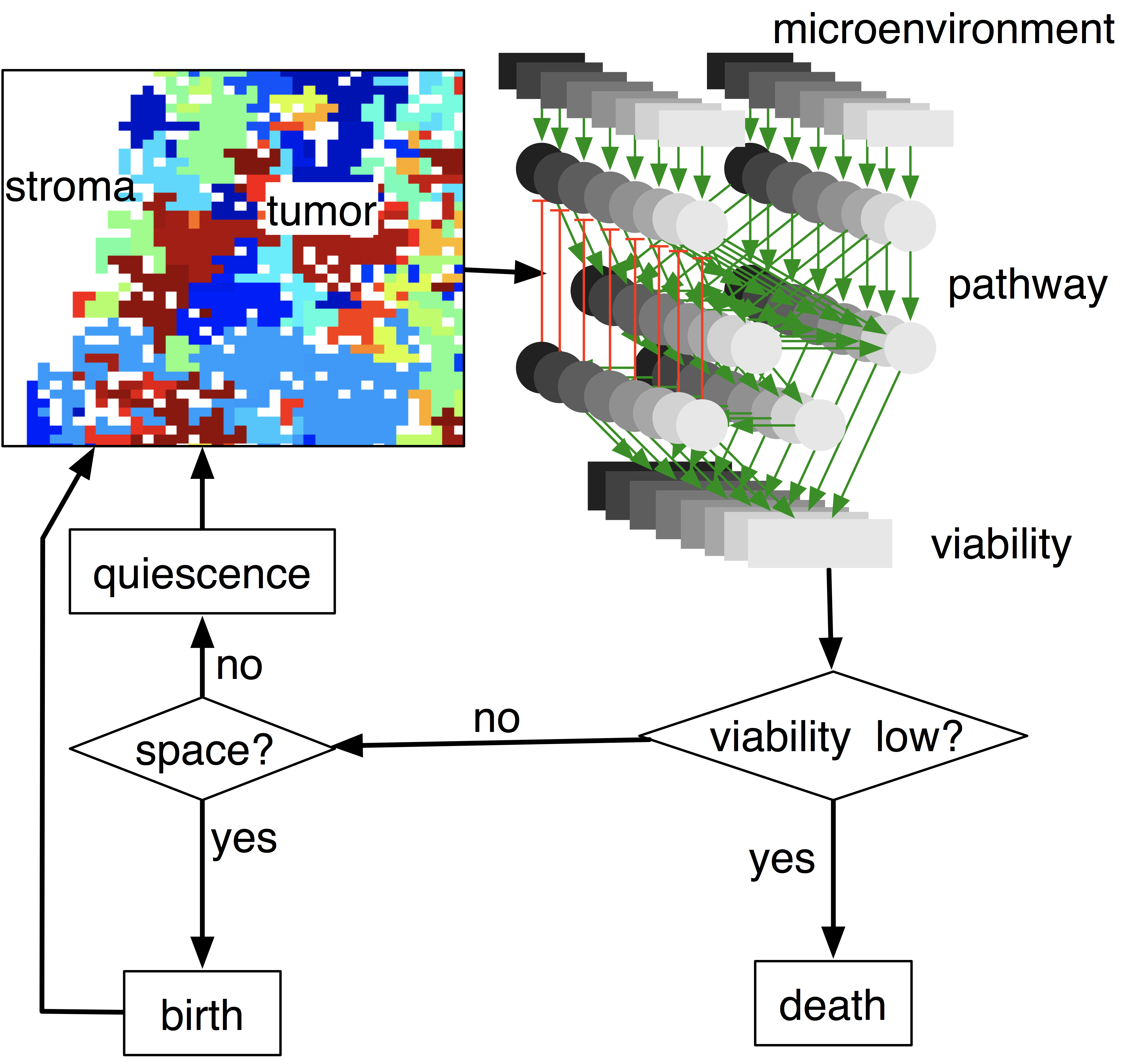

Supplement: S1 Fig — Each cell contains its own signaling network (calibrated network model, Fig 2A) and processes signaling to determine its viability. If the viability is low, the cell commits death with a probability of the viability. Otherwise, the cell waits for the next time step. If the cell viability is not too low, we check for an empty space in its nearest four neighbors (north, east, south, west from the cell). If there is an empty space, the cell divides with a probability of the viability. If there is no empty space, the cell becomes quiescent and waits for next time step. HCA, hybrid cellular automata. (TIFF) [file pbio.2002930.s001.tiff]

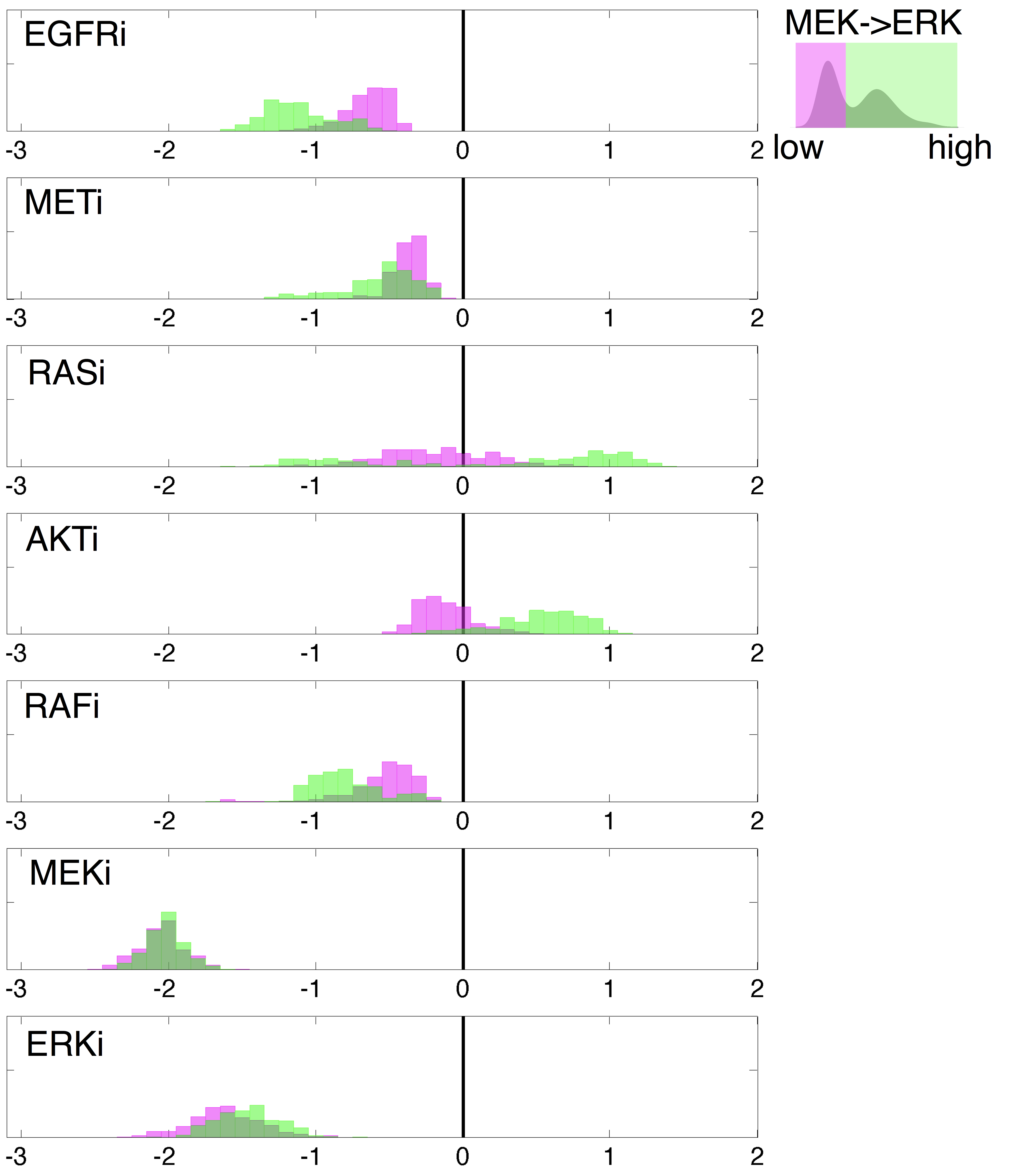

Supplement: S2 Fig — Then, distributions of treatment responses for each group were considered. The in silico cells with larger MEK-ERK weights seem to be more sensitive to both EGFRi and RAFi and are more resistant to AKTi than the ones with smaller MEK-ERK weights. AKT (PKB), protein kinase B; EGFR, epidermal growth factor receptor; ERK, extracellular receptor kinase; MEK, mitogen-activated protein kinase kinase; RAF, rapidly accelerated fibrosarcoma. (TIFF) [file pbio.2002930.s002.tiff]

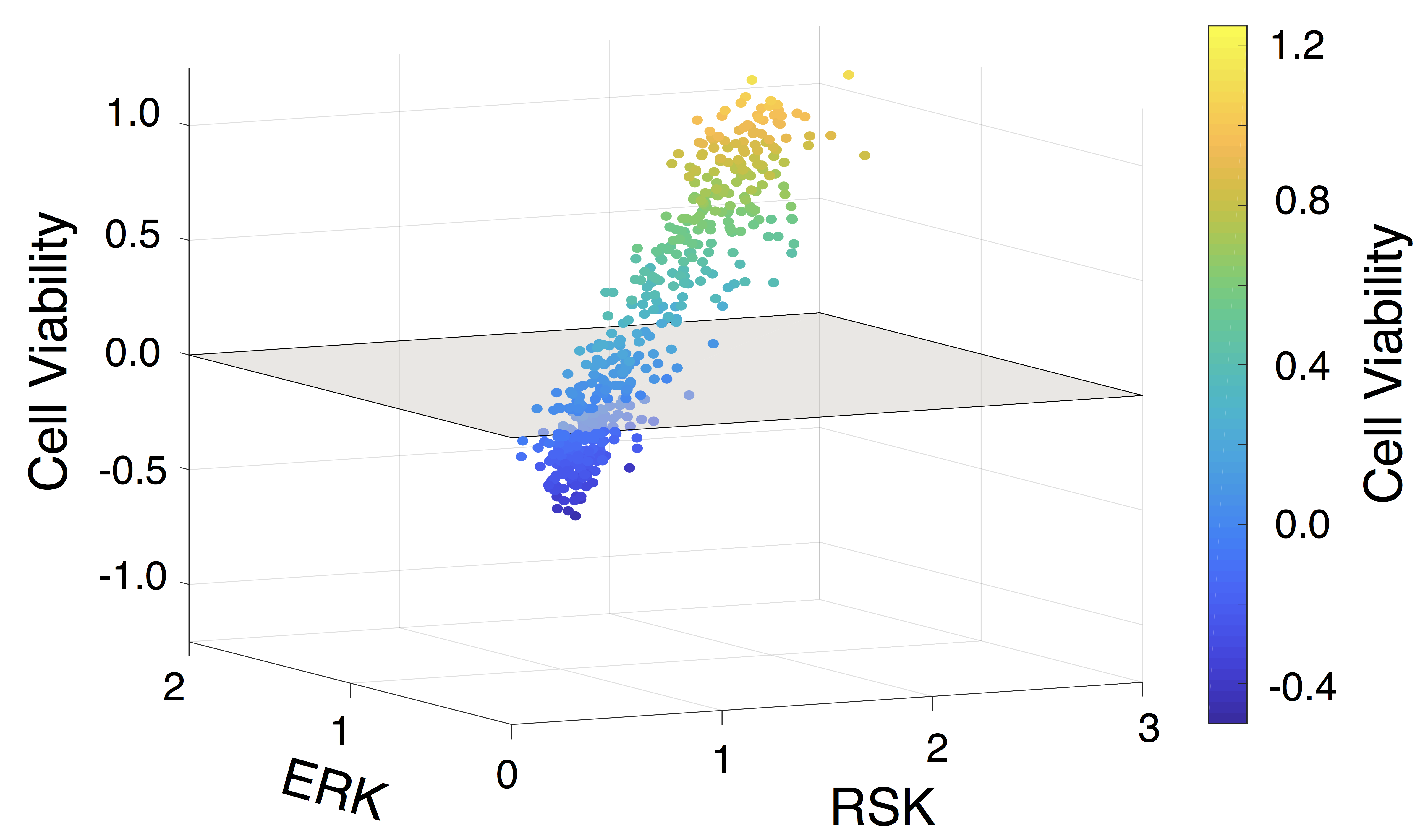

Supplement: S3 Fig — A scatter plot of relative cell viability (ratio of cell viability after treatment to cell viability before treatment, log 2 scale) as a function of relative activities of ERK and RSK (ratio of protein activity after treatment to protein activity before treatment, log 2 scale) is given. Color represents cell viability (blue: small and yellow: large). Gray plane indicates no change of cell viability. AKT (PKB), protein kinase B; ERK, extracellular receptor kinase; RSK, ribosomal S6 kinase. (TIFF) [file pbio.2002930.s003.tiff]

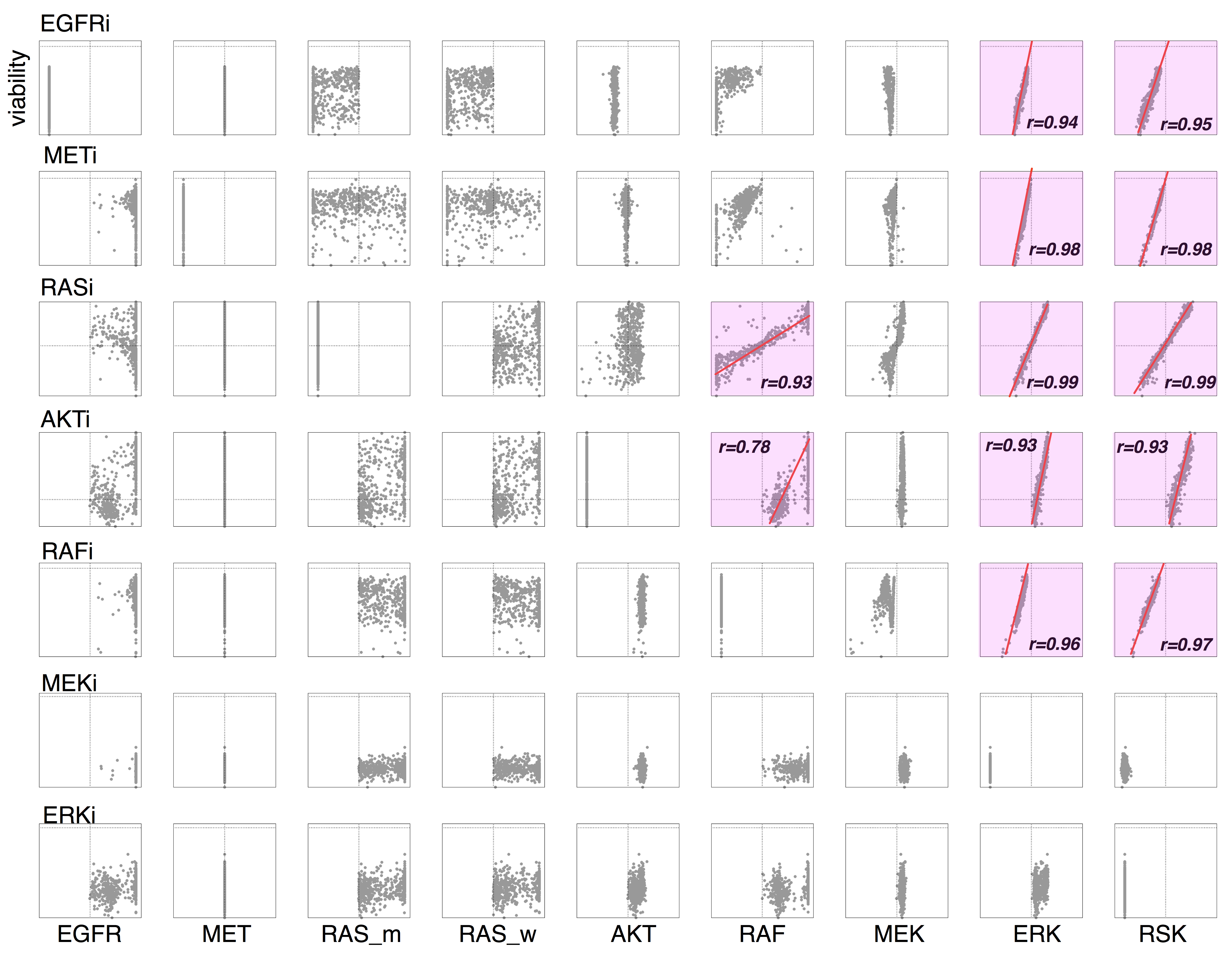

Supplement: S4 Fig — Scatter plots of cell viability changes (y axis) against individual protein activity changed (labeled on the bottom, EGFR, MET, RAS_m, RAS_w, AKT, RAF, MEK, ERK, RSK) after each monotherapy (labeled on the top of each row; first row: EGFRi; second row: METi; third: RAS_mi; fourth: AKTi; fifth: RAFi; sixth: MEKi; seventh: ERKi). Each dot represents an individual in silico cell. Dotted lines: no change; colored box: highly correlated relations; r: correlation coefficient. AKT (PKB), protein kinase B; EGFR, epidermal growth factor receptor; ERK, extracellular receptor kinase; MEK, mitogen-activated protein kinase kinase; MET (c-MET), tyrosine-protein kinase Met or hepatocyte growth factor receptor (HGFR); RAF, rapidly accelerated fibrosarcoma; RAS, rat sarcoma. (TIFF) [file pbio.2002930.s004.tiff]

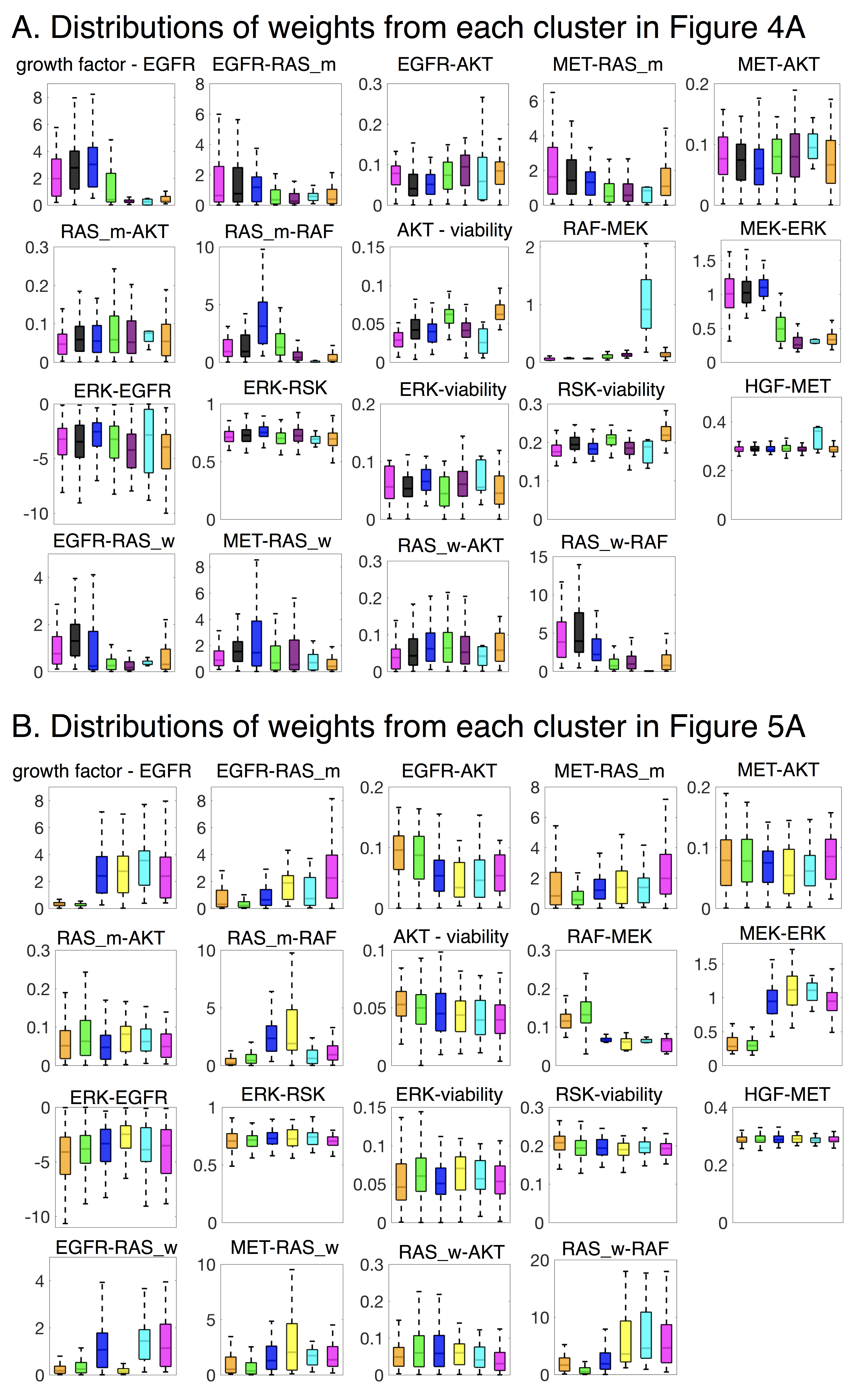

Supplement: S5 Fig — (A) Box plots of weights from each cluster in Fig 4A. Colors correspond to the different clusters in Fig 4A. (B) Boxplots of weights from each cluster in Fig 5A. Colors correspond to the different clusters in Fig 5A. (TIFF) [file pbio.2002930.s005.tiff]

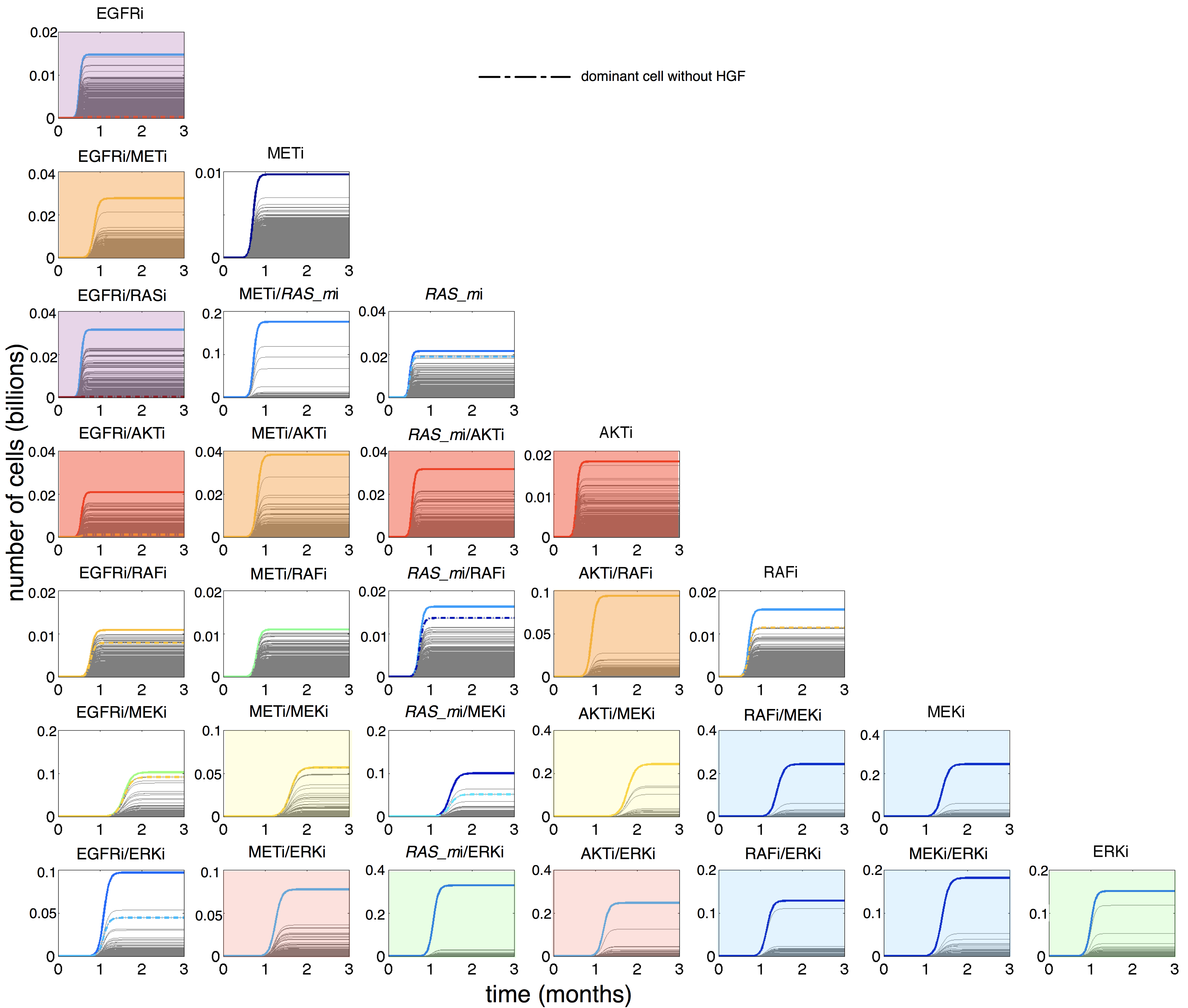

Supplement: S6 Fig — Color-shaded boxes indicate treatments selecting the same dominant clone. Compared with no-HGF treatment (Fig 6A), the treatments of EGFRi/MEKi, RAS_mi/MEKi, and EGFRi/ERKi select a different dominant cell (dashed-color line vs solid-color line). AKT (PKB), protein kinase B; corr, linear correlation; EGFR, epidermal growth factor receptor; ERK, extracellular receptor kinase; HGF, hepatocyte growth factor; MEK, mitogen-activated protein kinase kinase; MET (c-MET), tyrosine-protein kinase Met or hepatocyte growth factor receptor (HGFR); PI3K, phosphoinositide 3-kinase; RAF, rapidly accelerated fibrosarcoma; RAS, rat sarcoma. (TIFF) [file pbio.2002930.s006.tiff]

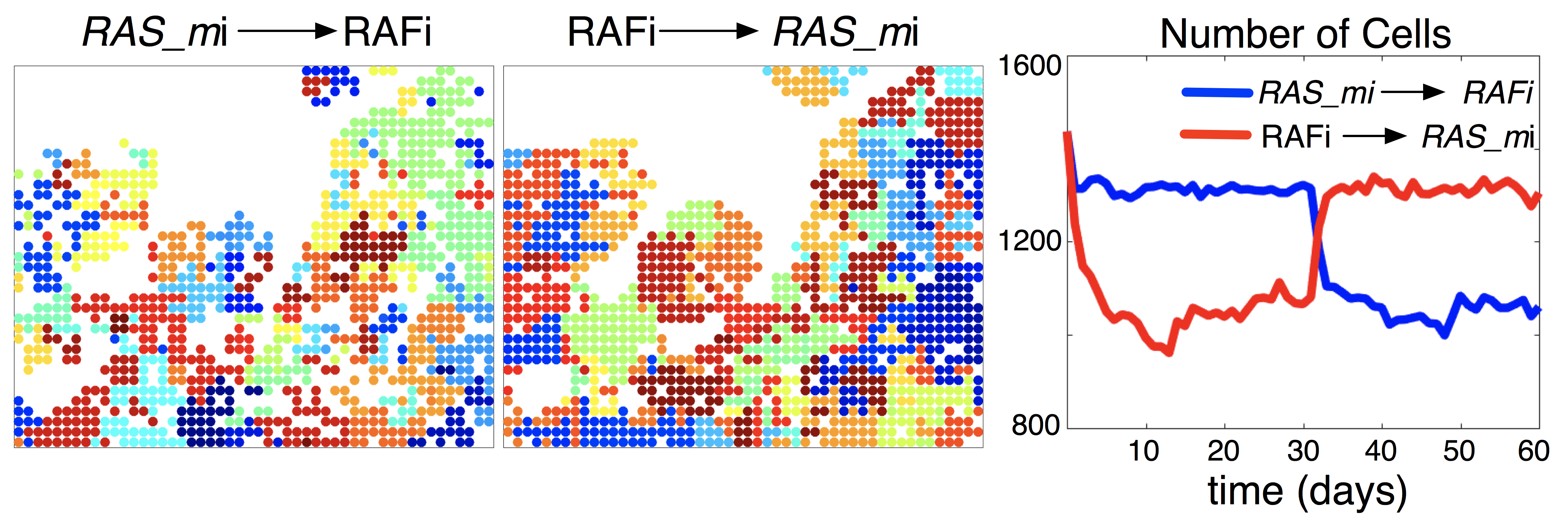

Supplement: S7 Fig — (A) Cell configuration after 30 days of RAS_mi first followed by 30 days of RAFi. Different cells are represented by different colors. (B) Cell configuration after 30 days of RAFi first followed by 30 days of RAS_mi. Different cells are represented by different colors. (C) Total number of cells over time; blue: RAS_mi → RAFi; red: RAFi → RAS_mi. HCA, hybrid cellular automata; RAF, rapidly accelerated fibrosarcoma; RAS, rat sarcoma. (TIFF) [file pbio.2002930.s007.tiff]

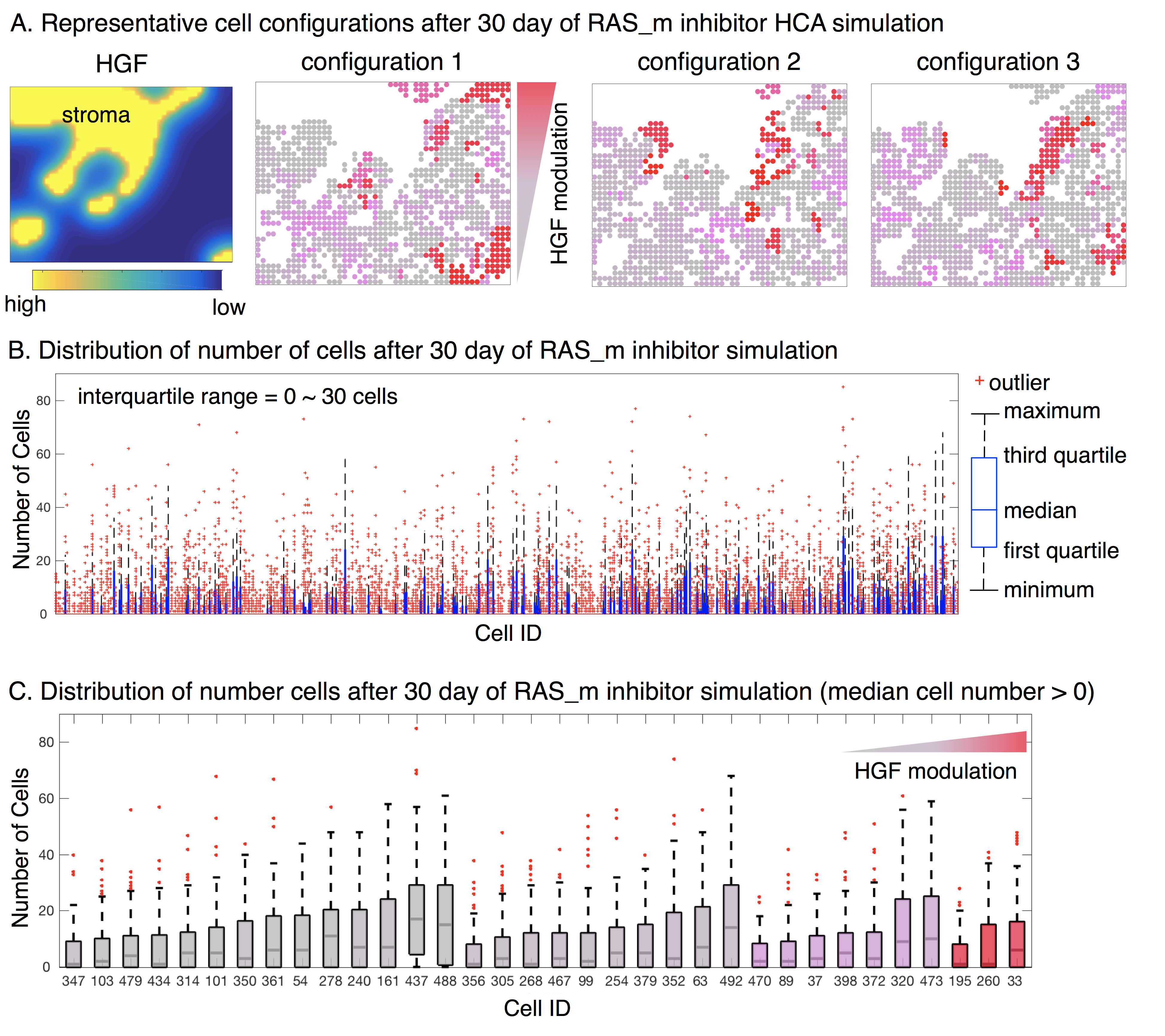

Supplement: S8 Fig — (A) Three different representative configurations of cells at time step 30. Color: HGF modulation, gray: no change of cell viability due to HGF stimulation; violet: significant increase of cell viability due to HGF. (B) Distribution of number of cells at time step 30. Boxplots of number of all of 500 cells at time step 30. “+” indicates outliers. The maximum difference of interquartile range is 30 cells (approximately 0.02% of total cell population). (C) Boxplots of the number of cells whose median cell numbers at time step 30 is greater than 0. Color: HGF modulation; gray: no change of cell viability due to HGF stimulation; violet: significant increase of cell viability due to HGF. HCA, hybrid cellular automata; HGF, hepatocyte growth factor; RAS, rat sarcoma. (TIFF) [file pbio.2002930.s008.tiff]

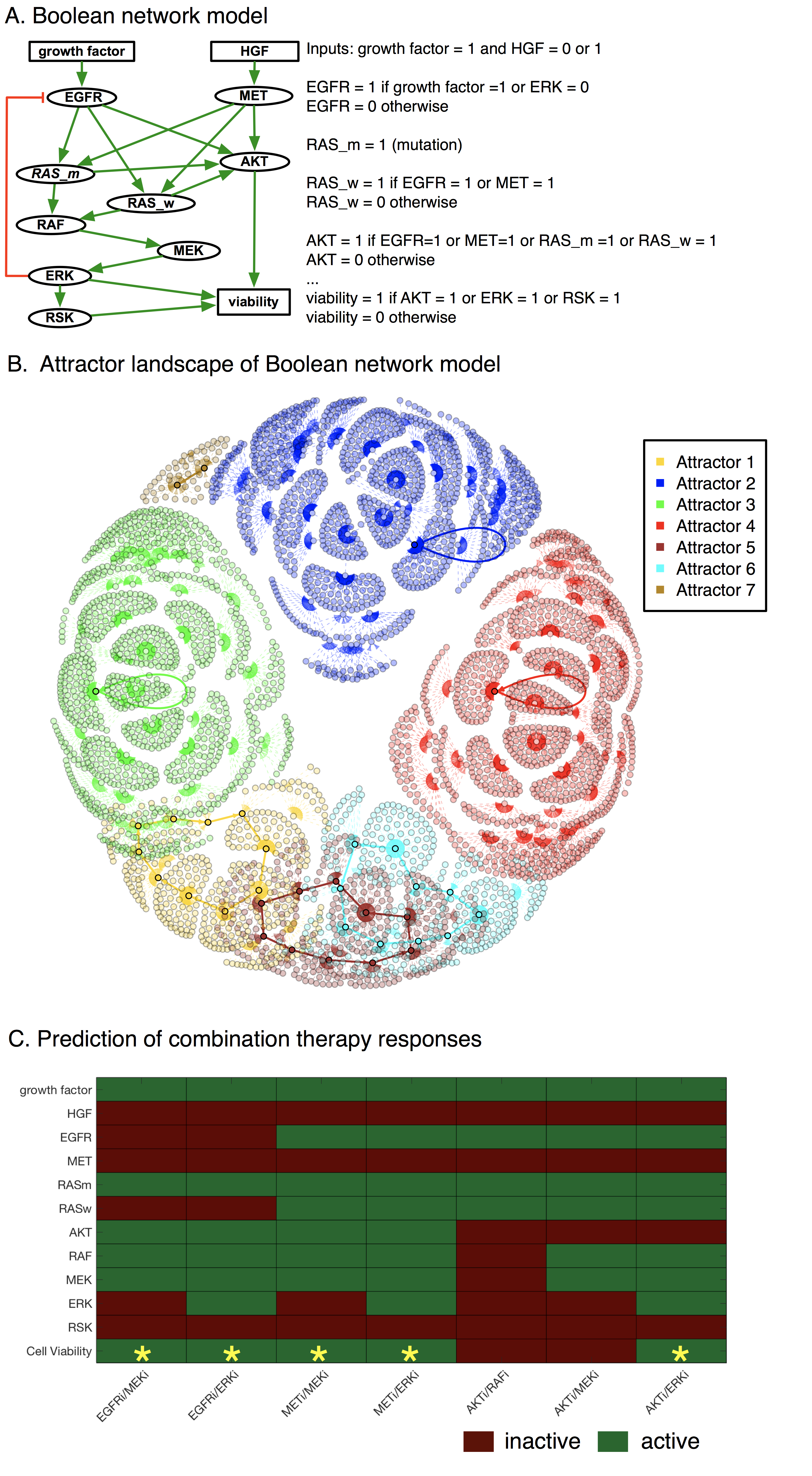

Supplement: S9 Fig — (A) Boolean network model. (B) Seven different attractors represented by different colors. (C) Active or inactive state of proteins and cell viability after various mono and combination therapies were applied. Yellow star: cell viability state that is inconsistent with experimental data (Figs 2 and 3). (TIFF) [file pbio.2002930.s009.tiff]

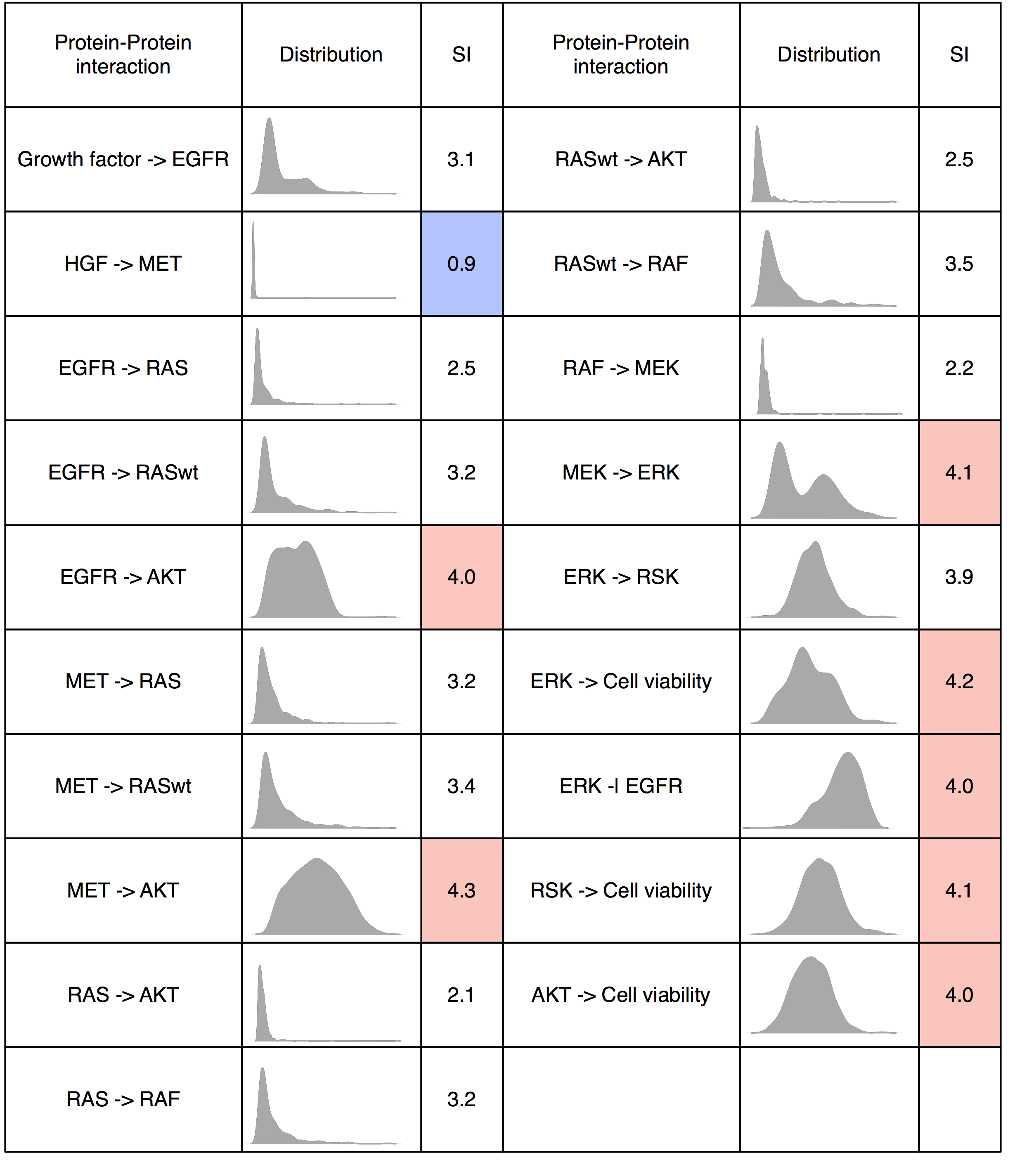

Supplement: S1 Table — Shannon index (SI) was also reported. A blue box indicates weights with the lowest Shannon index, while red boxes indicate weights with large Shannon index (>4.0). (TIFF) [file pbio.2002930.s012.tiff]
