# Supplementary material for: Cell signaling heterogeneity is modulated by both cell-intrinsic and -extrinsic mechanisms: An integrated approach to understanding targeted therapy
Source: PLoS Biol. 2018 Mar 9;16(3):e2002930. doi: 10.1371/journal.pbio.2002930 (PMC5844524; doi:10.1371/journal.pbio.2002930)
Supplement: S1 Text — RMSE, root-mean-squared-error. (DOCX) [file pbio.2002930.s010.docx]

Model calibration

A RMSE (root mean square error) was used to evaluate goodness of fit, where RMSE is defined as

$$\text{RMSE}=\sqrt{\frac{\sum_{i=1}^{m} \left(\overline{x_{i}}-y_{i}\right)^{2}}{m}},\text{ }$$

where $\overline{x_{i}}$ is a predicted value and $y_{i}$ is an experimentally observed value.
