# Supplementary material for: Cell signaling heterogeneity is modulated by both cell-intrinsic and -extrinsic mechanisms: An integrated approach to understanding targeted therapy
Source: PLoS Biol. 2018 Mar 9;16(3):e2002930. doi: 10.1371/journal.pbio.2002930 (PMC5844524; doi:10.1371/journal.pbio.2002930)
Supplement: S2 Text — We constructed an equivalent Boolean model of the signaling pathway (S9 Fig). In the model, each node has a binary value (1: active, on; 0: inactive, off). The behavior of each node is modeled as a sequence of discrete steps in a Boolean function defining the value of a node on the next step based on values of its neighbor nodes (S9A Fig). For all nodes except EGFR, a node will be active if at least one of its neighbors is active. The node EGFR will be active if either growth factor is active or ERK is inactive (inhibitory regulation of EGFR by ERK). Of note, RAS_m node is always active, representing RAS mutation in the cell line (A549) used in our experiments. Assuming both of the input nodes—(growth factor and HGF) and RAS_m—are always active, all possible initial states (210) are exhaustively simulated, using the R package BoolNet [88], until reaching attractors (steady states). The simulations converged on seven different attractors (S9B Fig). We then simulated seven different combination therapies that we tested in our experiments (Fig 3C). To simulate drug-induced inhibition, we made each target node constitutively inactive (e.g., EGFR = 0 for EGFRi; MET = 0 for METi; AKT = 0 for AKTi; ERK = 0 for MEKi; and RSK = 0 for ERKi). Two drug combinations result in an inactive viability state (S9C Fig, viability in red, AKTi/RAFi, AKTi/MEKi), which are consistent with both our modeling and experimental data (Fig 3C and Fig 4A). The Boolean network model predicts that other combinations are not effective (S9C Fig, active viability state in green), which are not consistent with both our model predictions and experimental data (yellow asterisks in S9C Fig vs Fig 3C). Taken together, these results suggest that this simple Boolean network is insufficient to recapitulate our experimental data. (DOCX) [file pbio.2002930.s011.docx]

**Boolean Network Model**

To investigate predictability of simpler models such as Boolean model, we constructed an equivalent Boolean model of the signaling pathway (Fig. S9). In the model, each node has a binary value (1: active, on, 0: inactive, off). The behavior of each node is modeled as a sequence of discrete steps in a Boolean function defining the value of a node on the next step based on values of its neighbor nodes (Fig. S9A). For all nodes except EGFR, a node will be active if at least one of its neighbors is active. The node EGFR will be active if either growth factor is active or ERK is inactive (inhibitory regulation of EGFR by ERK). Of note, *RAS_m* node is always active, representing RAS mutation in the cell line (A549) used in our experiments. Assuming both of the input nodes, (growth factor) and *RAS_m,* are always active all possible initial states ($2^{10}$) are exhaustively simulated, using the R package *BoolNet* (1), until reaching attractors (steady states). The simulations converged on seven different attractors (Fig. S9B). We then simulated seven different combination therapies that we tested in our experiments (Fig. 3C). To simulate drug-induced inhibition, we made each target node constitutively inactive (e.g., EGFR = 0 for EGFRi, MET = 0 for METi, AKT= 0 for AKTi, ERK = 0 for MEKi and RSK = 0 for ERKi, see experiments Fig. 2D).

Two drug combinations result in an inactive viability state (Fig. S9C, viability in red, AKTi/RAFi, AKTi/MEKi), which are consistent with both our modeling and experimental data (Fig.3C and Fig. 4A). The Boolean network model predicts that other combinations are not effective (Fig. S9C, active viability state in green), which are not consistent with both our model predictions and experimental data (yellow asterisks in Fig. S9C vs. Fig. 3C). Taken together, these results suggest that the Boolean model is insufficient to predict combination therapies.

1. Mussel C, Hopfensitz M, Kestler HA. BoolNet--an R package for generation, reconstruction and analysis of Boolean networks. Bioinformatics. 2010;26(10):1378-80.
